# Single-cell RNA-seq reveals novel regulators of human embryonic stem cell differentiation to definitive endoderm

**DOI:** 10.1186/s13059-016-1033-x

**Published:** 2016-08-17

**Authors:** Li-Fang Chu, Ning Leng, Jue Zhang, Zhonggang Hou, Daniel Mamott, David T. Vereide, Jeea Choi, Christina Kendziorski, Ron Stewart, James A. Thomson

**Affiliations:** 1 Morgridge Institute for Research, Madison, WI USA; 2 Department of Cell & Regenerative Biology, University of Wisconsin-Madison, Madison, WI USA; 3 Department of Molecular, Cellular, & Developmental Biology, University of California, Santa Barbara, CA USA; 4 Department of Statistics, University of Wisconsin, Madison, WI USA; 5 Department of Biostatistics and Medical Informatics, University of Wisconsin, Madison, WI USA; 6 Present address: Genentech, Inc., South San Francisco, CA USA; 7 Present address: Department of Cell Biology, Harvard Medical School, Boston, MA USA

**Keywords:** Single-cell RNA-seq, Embryonic stem cells, Mesendoderm, *Brachyury*, Definitive endoderm, Wave-Crest, SCPattern, *KLF8*, CRISPR/Cas9

## Abstract

**Background:**

Human pluripotent stem cells offer the best available model to study the underlying cellular and molecular mechanisms of human embryonic lineage specification. However, it is not fully understood how individual stem cells exit the pluripotent state and transition towards their respective progenitor states.

**Results:**

Here, we analyze the transcriptomes of human embryonic stem cell-derived lineage-specific progenitors by single-cell RNA-sequencing (scRNA-seq). We identify a definitive endoderm (DE) transcriptomic signature that leads us to pinpoint a critical time window when DE differentiation is enhanced by hypoxia. The molecular mechanisms governing the emergence of DE are further examined by time course scRNA-seq experiments, employing two new statistical tools to identify stage-specific genes over time (SCPattern) and to reconstruct the differentiation trajectory from the pluripotent state through mesendoderm to DE (Wave-Crest). Importantly, presumptive DE cells can be detected during the transitory phase from *Brachyury (T)*^*+*^ mesendoderm toward a *CXCR4*^*+*^ DE state. Novel regulators are identified within this time window and are functionally validated on a screening platform with a *T-2A-EGFP* knock-in reporter engineered by CRISPR/Cas9. Through loss-of-function and gain-of-function experiments, we demonstrate that *KLF8* plays a pivotal role modulating mesendoderm to DE differentiation.

**Conclusions:**

We report the analysis of 1776 cells by scRNA-seq covering distinct human embryonic stem cell-derived progenitor states. By reconstructing a differentiation trajectory at single-cell resolution, novel regulators of the mesendoderm transition to DE are elucidated and validated. Our strategy of combining single-cell analysis and genetic approaches can be applied to uncover novel regulators governing cell fate decisions in a variety of systems.

**Electronic supplementary material:**

The online version of this article (doi:10.1186/s13059-016-1033-x) contains supplementary material, which is available to authorized users.

## Background

The three primary germ layers composed of lineage-specific progenitors are critical for the establishment of the embryonic body plan [1–4]. Directional differentiation protocols have efficiently driven human pluripotent stem cells into progenitor populations mimicking those of the embryonic ectoderm, mesoderm, endoderm, and extraembryonic lineages [5–18]. However, it is not fully understood how individual embryonic stem (ES) cells exit the pluripotent state and give rise to lineage-specific progenitors.

Among the three primary germ layers, the definitive endoderm (DE) is the internal layer of the embryonic gut, formed by the recruitment of epiblast cells through the primitive streak. The DE cells give rise to a variety of functional specialized epithelial cell types that line the developing gut tube and contribute to vital organs or tissues such as the lungs, trachea, esophagus, liver, stomach, intestine, thyroid, thymus, and pancreas [1–3]. These endoderm-derived organs support indispensible functions in adults, such as gas exchange in respiration, mechanical and chemical digestion, and blood glucose homeostasis and detoxification. Therefore, human pluripotent stem cell-derived DE cells are an instrumental resource for regenerative medicine [7, 9, 10, 15, 18, 19]. However, the factors governing the transition from epiblast-derived precursors to the DE state is not fully understood.

Mesendoderm represents a transient state, composed of a migratory cell population emerging from the primitive streak. Its emergence is accompanied by the activation of the transcription factor *Brachyury (T)*, which marks the onset of gastrulation [2, 11, 20]. As gastrulation continues, mesendoderm contributes to mesoderm or DE. Once the lineage decision is segregated, *T* expression appears to be continually associated with certain mesodermal derivatives but not DE derivatives [11, 21, 22]. This represents a key developmental juncture when cell fate decisions have been made from a broad multi-potent state (mesendoderm) towards a more restricted state (definitive endoderm). Therefore, we designed our scRNA-seq experiments to detect signals that could promote DE differentiation and then followed up these experiments with a detailed time course to identify the critical time window in which mesendoderm transitions to the DE state.

Standard methods for transcriptome-wide profiling of differentiation involves the collection of thousands to millions of cells for deep sequencing (bulk RNA-seq) at one or several time points. With this approach, cellular heterogeneity cannot be resolved since variably expressed genes will be averaged or – if exclusively expressed in rare cells – completely missed. Single-cell RNA-seq (scRNA-seq), on the other hand, is able to characterize cell-to-cell variation and reveal transcriptomic signatures unique to individual cells [23–25]. Such analyses can provide novel insights into the responses to extrinsic signals and reveal intrinsic factors that control cell fate decisions. These insights can then guide the genesis of more sophisticated differentiation protocols and quality control assays.

To understand the distinctions between DE cells and the other lineage-specific progenitors, we examined their transcriptomes by scRNA-seq. Our analysis revealed a DE-specific signature that is enriched for NODAL and WNT signaling pathways as well as metabolism-related gene expression. The latter category of genes led us to define a time window in which hypoxia could enhance DE marker expression. Based on this observation, we hypothesized that the emergence of nascent DE cells occurs as soon as two days post differentiation from the pluripotent state.

Compared to single time point experiments, time course scRNA-seq has the potential to reveal detailed cell state transitions [26–28]. To pinpoint the exact timing of DE cell emergence, we profiled the transition of single human ES cells to mesendoderm then to the DE state over four days of differentiation. To analyze the transition at the single-cell level, we developed two novel statistical tools. First, SCPattern [29] is used to identify stage-specific genes over time; and second, Wave-Crest is used to reconstruct the differentiation trajectory from the pluripotent state through mesendoderm to DE. Based on this high-resolution temporal reconstruction, we detected presumptive DE cells characterized with *CXCR4* and *SOX17* expression as early as 36 h post differentiation. Focusing on this time point, Wave-Crest identified candidate genes that could function as pioneer regulators governing the transition from mesendoderm to the DE state.

Owing to known technical variability and stochastic expression in single-cell gene expression measurements [30–33], rigorous functional validation of scRNA-seq analyses is essential. In order to specifically validate our analysis, we engineered a *T-2A-EGFP* reporter ES cell line by CRISPR/Cas9-mediated knock-in. Of all the candidate genes tested, we found that siRNA knockdown of *KLF8* rendered one of the most overt delays in differentiation. A converse gain-of-function experiment demonstrated that *KLF8* plays a previously unrecognized role during the transition from a *T*^*+*^ state to a *CXCR4*^*+*^ DE state. Our results reveal that elevated levels of *KLF8* enhance expression of DE markers but not mesodermal genes, suggesting that *KLF8* acts specifically on the transition from mesendoderm to DE but not to mesoderm. Altogether, our study reinforces the importance of combining single-cell analysis and genetic approaches. We believe this strategy could be directly applied to other lineages during any differentiation paradigm to examine cell fate decisions.

## Results

### scRNA-seq reveals a unique endoderm progenitor signature

**Fig. 1:**
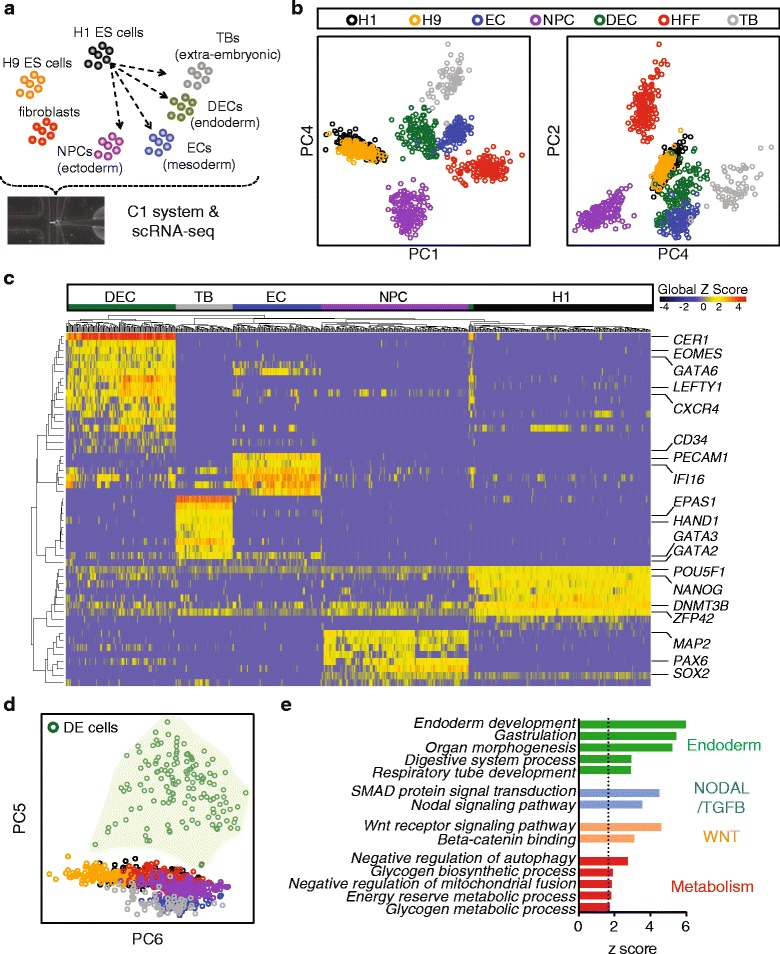
Snapshot scRNA-seq analysis of human ES-derived progenitors. **a** Schematics of experimental strategy. Human ES-derived lineage-specific progenitors were profiled at the single-cell resolution. **b** Principal component analysis (PCA) of all the cell types profiled. Shown are PC4 vs. PC1 and PC2 vs. PC4. **c** Hierarchical clustering analysis of progenitors differentiated from H1 cells with selected lineage-specific markers shown on the *right*. **d** PCA of all the cell types profiled, shown are PC5 vs. PC6. The *light green shade* highlights all the single DE cells. **e** Enrichment analysis of PC5 shown in (**d**). Bar graph shows the significant z scores of selected GO terms. Summary on collections of GO terms are shown on the *right. Dashed line* indicates statistical significant threshold at z score = 1.62 (one tailed *p* value <0.05). *NPC* neuronal progenitor cell, *DEC* definitive endoderm cell, *EC* endothelial cell, *TB* trophoblast-like cell, *HFF* human foreskin fibroblasts

To begin investigating lineage-specific transcriptomic features at single-cell resolution, we performed a cohort of scRNA-seq experiments profiling snapshots of lineage-specific progenitor cells differentiated from H1 human ES cells using our established differentiation protocols, all adapted to chemically-defined culture conditions [17, 20, 34, 35]. In order to obtain a high purity of lineage-specific progenitors, cells were enriched by fluorescence-activated cell sorting (FACS) with their respective markers (see details in “Methods” and Additional file 1: Figure S1). Progenitors differentiated from human ES cells included neuronal progenitor cells (NPCs, ectoderm derivatives, n = 173), DE cells (endoderm derivatives, n = 138), endothelial cells (ECs, mesoderm derivatives, n = 105), and trophoblast-like cells (TBs, extraembryonic derivatives, n = 69). Single undifferentiated H1 (n = 212) and H9 (n = 162) human ES cells and human foreskin fibroblasts (HFFs, n = 159) were also included as controls. In total, 1018 single cells were analyzed in this cohort of experiments (Fig. 1a and Additional file 1: Figure S1).

To elucidate the distinctions between different lineages, we conducted bulk-projected principal component analysis (PCA), which projects individual cells on bulk RNA-seq defined principal component (PC) axes (see details in “Methods”). The majority of the single cells cluster according to their respective developmental lineages (Fig. 1b). The tight clusters of overlapped H1 and H9 single cells indicated the relative homogeneity of human ES cells compared to the rest of the progenitors. NPCs (ectoderm), TBs (extraembryonic), and HFFs (fibroblasts) were distantly positioned on the PCA plot while ECs and DE cells showed some overlapped domains, suggesting a closer lineage relationship. This result is consistent with the notion that mesoderm and DE cells may arise from a common progenitor pool during development and differentiation [20, 36, 37]. Hierarchical clustering analysis of key lineage markers further demonstrates distinct but rather uniform expression within each progenitor state (Fig. 1c). Specifically, all of the single undifferentiated H1 cells uniformly expressed high levels of pluripotency markers including *POU5F1*, *NANOG*, *DNMT3B*, and *ZFP42 (REX1)*. By contrast, NPCs are enriched for the expression of *SOX2*, *PAX6*, and *MAP2*; ECs are enriched for *PECAM1* and *CD34*; TBs are enriched for *GATA3* and *HAND1*; and DE cells are enriched for *CER1*, *EOMES*, *GATA6*, *LEFTY1*, and *CXCR4* (Additional file 2: Table S1). These analyses indicate that scRNA-seq of the progenitors is competent to reveal lineage-specific transcriptomic features.

The bulk-projected PCA shows that the majority of variation could be captured by the first five PCs (Additional file 1: Figure S2). Interestingly, PC5 clearly separates DE cells from all the other progenitors (Fig. 1d). This result suggests that PC5 gene loadings are a signature exclusively exhibited by differentiating DE cells. We also observed single DE cells distributed along the PC5 axis, indicating that this gene loading captured a heterogeneous or asynchronous pool of DE cells. Moreover, DE cells showed the greatest heterogeneity compared to the other three types of progenitors by correlation analysis (Additional file 1: Figure S2). To determine the signaling pathways associated with the DE signature, we performed Gene Ontology (GO) analysis utilizing the Allez algorithm, which used absolute gene loadings to weight gene-specific contribution to PC5 (see “Methods”) [38]. Allez enrichment analysis identified endoderm development, organ morphogenesis, NODAL signaling pathway, regulation of WNT receptor signaling pathway, and energy reserve metabolic processes among the significantly enriched GO terms (Fig. 1e and Additional file 3: Table S2). While it is well established that both NODAL and WNT signaling are crucial for endoderm development [1–3, 11], little is known about how the metabolic state could influence DE differentiation. Based on these analyses, we investigated whether manipulating the metabolic conditions could impact DE differentiation.

### Acute hypoxic treatment enhanced DE differentiation

It has been previously shown that lowering oxygen tension can reduce oxidative stress, shifting metabolic fueling pathways from oxidative phosphorylation to glycolysis to aid in maintaining pluripotency and reprogramming [39, 40]. We therefore set out to measure the impact of lowering oxygen concentration in the cell culture microenvironment during the differentiation toward DE (see “Methods”). We chose to monitor the expression of Chemokine (C-X-C Motif) receptor 4 (*CXCR4*) in live cells as a surrogate marker for DE differentiation because: (1) *CXCR4* expression is specific to DE but absent in the visceral endoderm compartment at the late primitive streak stage (~E7.5) mouse embryo [41]; and (2) human ES-derived CXCR4^+^ cells have been shown to display hallmarks of DE [7, 15, 42].

**Fig. 2:**
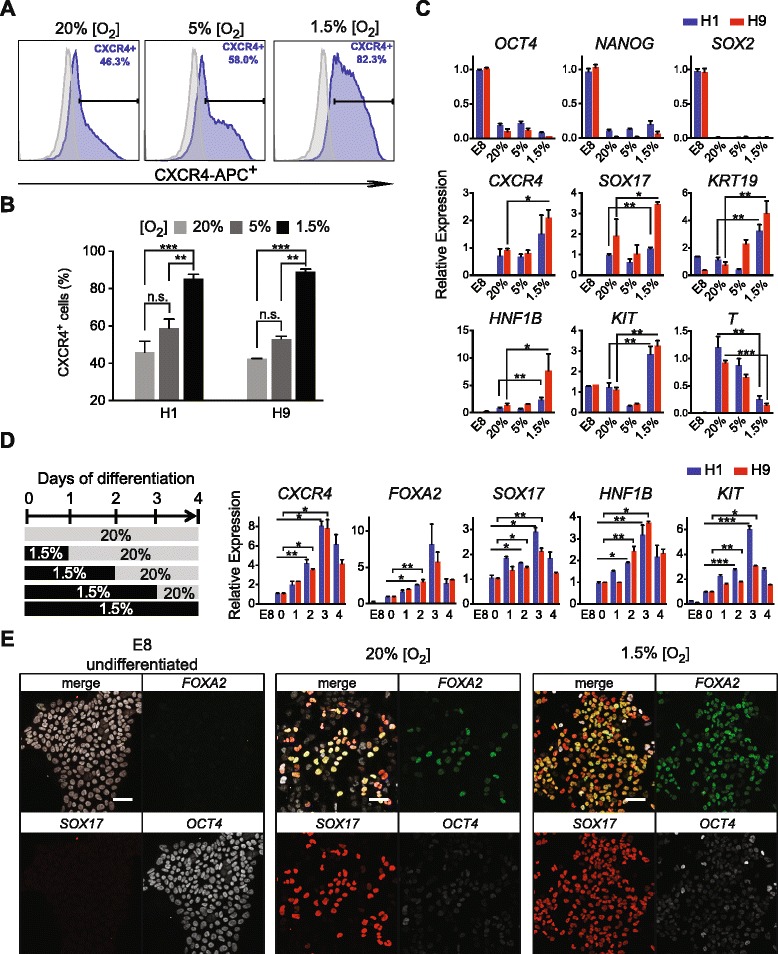
Acute hypoxic treatment enhanced DE formation. **a** FACS analysis of anti-CXCR4 staining of H1 cells differentiated for three days towards (*blue population*) under various hypoxia conditions. Undifferentiated H1 cells were gated as negative controls (*gray populations*). The *x-axis* indicates the APC channel. **b** Summary of the percentages of CXCR4^+^ cells (with various oxygen concentrations) from H1 or H9 differentiation for three days toward DE cells. **c** QPCR analysis of experiments performed in (**b**). All expression levels were first normalized to endogenous GAPDH. For pluripotency markers (*upper panels*), samples were normalized to undifferentiated H1 or H9 samples, which were arbitrarily set to 1. For other markers (*mid* and *lower panels*), samples were normalized to the 20 % O_2_ samples, which were arbitrarily set to 1. **d** *Left panel*, schematics of various lengths of hypoxic treatment. *Right panel*, qPCR analysis at day four of differentiation for H1 or H9 cells. The *x-axis* indicates the number of days treated with 1.5 % O_2_ as indicated in the *left panel*. Samples were normalized to those from normoxia, which were arbitrarily set to 1. **e** Confocal images of OCT4, FOXA2, and SOX17 immunofluorescence staining at day two of differentiation under 20 % or 1.5 % O_2_. Scale bars = 50 μm. All data are mean ± S.D. ***p <0.001; **p <0.01; *p <0.05, by one-tailed t-test. *n.s.* no significant difference

At three days of differentiation, H1 or H9 ES cells cultured in severe hypoxia (1.5 % O_2_) significantly increased the percentage of CXCR4^+^ cells by FACS analysis compared to hypoxia (5 % O_2_) or normoxia (20 % O_2_) conditions (Fig. 2a and b). The percentages of cells co-expressing CXCR4 and SOX17 also increased in 1.5 % O_2_ condition (Additional file 1: Figure S3). Marker studies by quantitative real-time PCR (qPCR) analysis confirmed that the expression of pluripotency genes *POU5F1*, *NANOG*, and *SOX2* were effectively downregulated in all conditions (Fig. 2c)*.* Importantly, key DE markers *CXCR4*, *SOX17*, *HNF1B*, *KIT*, and *KRT19* were all significantly upregulated in 1.5 % O_2_ but not in 5 % O_2_, compared with normoxic conditions. Interestingly, hypoxic conditions also significantly suppressed *T* expression, which is a pan-mesendoderm marker whose expression precedes DE marker expression (Fig. 2c).

While differentiating human ES cells with 1.5 % O_2_ has a profound effect on the induction of DE markers, we also observed that proliferation decreased and cell death increased after prolonged hypoxic differentiation (Additional file 1: Figure S3). Thus, we speculate that 1.5 % O_2_ treatment stimulates the emergence of nascent DE cells but may not be beneficial for long-term proliferation and maturation. To determine the critical timing when the effects of severe hypoxia could take place, we performed acute-hypoxia treatment in our differentiation protocols (Fig. 2d). In this set of experiments, H1 or H9 ES cells were first differentiated in 1.5 % O_2_ environment for various lengths (zero, one, two, three, or four days of differentiation) and then switched back to normoxic conditions for up to four days of differentiation (Fig. 2d). Consistently, qPCR analysis showed that continuous hypoxic treatment throughout the first three days of differentiation resulted in substantially enhanced expression of key DE markers, such as *CXCR4*, *FOXA2*, *SOX17*, *HNF1B*, and *KIT* (Fig. 2d). Remarkably, the marker expression showed significant upregulation within just two days of hypoxic treatment. Immunofluorescence staining of SOX17 and FOXA2 confirmed areas with an increased number of SOX17^+^ and FOXA2^+^ cells within two days of severe hypoxic differentiation condition compared to normoxia control (Fig. 2e). These results corroborate a recent mouse ES cell study in which hypoxia culture facilitated DE differentiation [43]. Most importantly, this observation suggests that the birth of nascent DE cells is a well-timed event. Intervention of this process by enhancing factors (in this case, with severe hypoxia) allows us to close in on the key moments in which DE cells become specified from their mesendoderm precursors. These results motivated us to closely examine the transition from mesendoderm to DE state at a higher temporal resolution.

### Reconstruction of temporal single-cell states identifies regulators for nascent DE cell differentiation

**Fig. 3:**
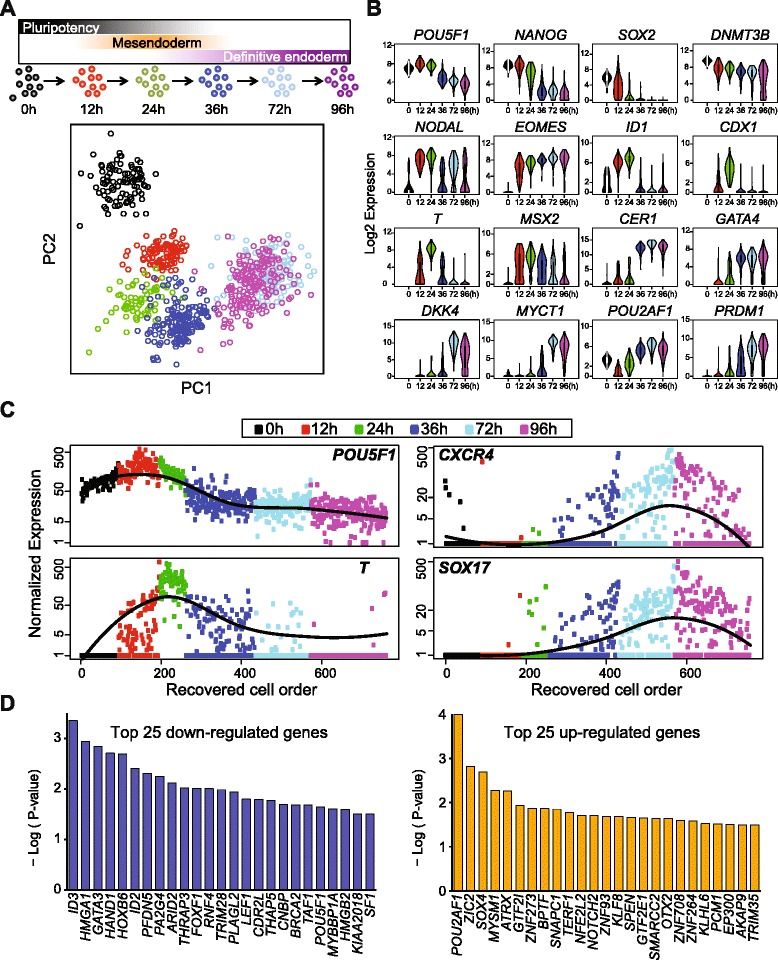
Time course scRNA-seq data analysis and reconstructing DE differentiation trajectory. **a** *Upper panel*, schematics of experimental strategy illustrating time points of scRNA-seq sampling along the differentiation from pluripotent state though mesendoderm to DE cells. *Lower panel*, PCA of scRNA-seq data, shown is PC1 vs. PC2. **b** Violin plots of selected stage-specific markers identified by SCPattern analysis. The *y-axis* indicates normalized expression value, Log2(expected count + 1). The *x-axis* indicates time points of sampling. **c** Reconstructed single-cell order by Wave-Crest. Shown are four of the eight genes used for temporal reconstruction. The *x-axis* indicates cells following Wave-Crest recovered cell order. The *y-axis* indicates normalized expression value. Fitted lines of gene-specific expression are shown in *black*. **d** Top 25 upregulated or downregulated genes identified by Wave-Crest at 36 h of differentiation. The *x-axis* indicates gene symbol, the *y-axis* indicates the significance value, -Log (*p* value) of each gene

To precisely pinpoint the staging and timing during DE emergence, we performed scRNA-seq at time points along the differentiation protocol to produce DE cells from human ES cells (see “Methods”). A total of 758 single cells were captured and profiled by scRNA-seq at 0, 12, 24, 36, 72, and 96 h of differentiation (Fig. 3a). PCA revealed that single cells from each time point along the differentiation course occupied a unique dimensional space, indicating a robust directional differentiation (Fig. 3a and Additional file 1: Figure S4). However, we noticed overlapping domains between single cells collected from 72 and 96 h of differentiation, indicating a similar transcriptome profile (see “Discussion”).

To further characterize the genes participating in each stage of differentiation, we performed differential expression analysis by SCPattern, which identifies significantly upregulated or downregulated genes between each pair of adjacent sampling time points (see “Methods”). SCPattern is a novel algorithm developed for differential expression analysis on time course scRNA-seq data [29]. Existing differential expression tools developed for bulk RNA-seq make distributional assumptions that are inappropriate for scRNA-seq data. In general, for a given gene, the bulk RNA-seq methods usually assume expression values within a biological condition follow a uni-modal distribution such as negative binomial distribution [44–48] or Poisson distribution [49]. However, such assumptions are often violated in scRNA-seq data due to the present of sub-populations and technical dropouts [50, 51]. SCPattern makes no parametrical assumptions on the distribution of the single-cell gene expression values; instead, it performs non-parametric tests based on a Kolmogorov–Smirnov statistic and is able to detect various types of changes over multiple ordered conditions [29].

At 12 h of differentiation, the majority of the cells responded to BMP4, Activin A, and CHIR 99021 (small molecule used as a WNT signaling agonist) treatment by robustly expressing *NODAL*, *EOMES*, and *ID1*. At 24 h of differentiation, a second wave of genes exhibited high levels of expression such as *T*, *MSX2*, and *CDX1*, all indicating a transition of the cells towards a primitive streak state. At 36 h of differentiation, the level of *T* transcripts rapidly decreased, characterized by upregulation of early DE-specific genes, such as *CER1* and *GATA4*. At 72 h of differentiation, the majority of the cells expressed endogenous *DKK4* and *MYCT1*. Key DE markers continue to be expressed at high levels among single cells at the 96-h time point, including *EOMES*, *CER1*, *GATA4*, *PRDM1*, and *POU2AF1,* indicating that cells progressed toward the DE state [52, 53] (Fig. 3b and Additional file 4: Table S3).

At any time point during a differentiation protocol, each sampled cell is not necessarily identical to the others; likely a result of differences in the cell cycle and the local microenvironment of the differentiating cells. We capitalized on the asynchronous nature of the cells to reconstruct a single-cell order following the differentiation trajectory toward DE. By reconstructing this single-cell transcriptomic order, we hoped to identify novel regulators whose expression could mediate the transition from mesendoderm toward a DE state. We devised a novel statistical tool, Wave-Crest, to reorder single cells according to the expression of key gene markers. The cell order reconstruction step of Wave-Crest takes a group of genes of interest and aims to recover a smooth expression profile along time for each of the genes in consideration. To do so, Wave-Crest implements a constrained extended nearest-insertion (ENI) algorithm to reorder cells within each time point utilizing boundary information from other time points. In particular, if a cell’s expression profile is closer to the cells from the previous (next) time point, the cell will be placed in an earlier (later) position in the reconstructed order. The reordering is under the constraint that cells from different collection times are not allowed to be mixed in the recovered order. After ENI, Wave-Crest utilizes the 2-opt algorithm to further refine the cell order (see “Methods”) [54]. When applied Wave-Crest to the scRNA-seq DE differentiation time course data, we selected the genes of interest by combining empirical results from SCPattern and prior knowledge [1, 2, 5, 7, 9, 20]. Our reconstruction focus on markers representing the pluripotent, mesendodermal, and DE states to build a directional reordering of single cells without characterizing the branching structure of single cells (Additional file 1: Figure S5). In particular, pluripotency marker *POU5F1* gradually decreased over the course of 96 h of differentiation, whereas mesendoderm marker *T* expression first peaked at 24 h and gradually decreased at 36–72 h of differentiation (Fig. 3c, left panel). Remarkably, in the reconstructed order, *CXCR4* and *SOX17* both showed a subtle but significant upregulation as early as 36 h of differentiation and continued to increase in later time points (Fig. 3c, right panel). Importantly, in the recovered cell order at the 36-h time point, *CXCR4*^*+*^ and *SOX17*^*+*^ single cells appear later and are almost mutually exclusive to the *POU5F1*^*high*^ and *T*^*high*^ cells, indicating our reconstructed cell order is indeed aligned with the differentiation trajectory toward a DE fate (Additional file 1: Figure S5). These results also suggest that the presumptive DE transcriptional program begins between 24 and 36 h, which is surprisingly early considering most established human pluripotent stem cell protocols typically consider DE cells to emerge around days 4 or 5 of differentiation [7, 11, 15, 42, 52].

The second step of Wave-Crest involves application of polynomial regression models to identify genes whose expression profile best fits this reconstructed differentiation trajectory, a strategy we call “fishing” (see “Methods”). We focused on fishing genes at 36 h of differentiation because this appears to be the transition time characterized with a steep downregulation of *POU5F1* and *T* as well as upregulation of *CXCR4* and *SOX17* (Fig. 3c and Additional file 1: Figure S5). Wave-Crest extracted the reconstructed cell order from the 172 cells collected at 36 h of differentiation and then fished against a curated list of transcriptional regulators (Additional file 5: Table S4). The top-fished genes were defined as the genes which had small fitting error in the polynomial regressions. Permutation tests were applied to infer the significance. These top-fished genes were then classified into upregulated and downregulated groups by the coefficient sign of gene-specific slope fitting. The top-fished gene list included known markers for mesendoderm or mesoderm (downregulated genes), such as *GATA3* (No. 3), *HAND1* (No. 4), *FOXF1* (No. 11), *LEF1* (No. 15), and markers for DE specification (upregulated genes), such as *SOX4* (No. 3) and *OTX2* (No. 18), further demonstrating the power of Wave-Crest reconstruction (Fig. 3d and Additional file 6: Table S5)*.* We reasoned that the top upregulated genes are likely to promote the transition from mesendoderm to DE (Fig. 3d). To test these genes, a reporter was devised to measure the effect of manipulation of candidate genes during the transition from a *T*^*+*^ state to a *CXCR4*^*+*^ state in live cells.

### *H9-T-2A-EGFP* reporter line is a robust tool to monitor the mesendoderm to DE transition

**Fig. 4:**
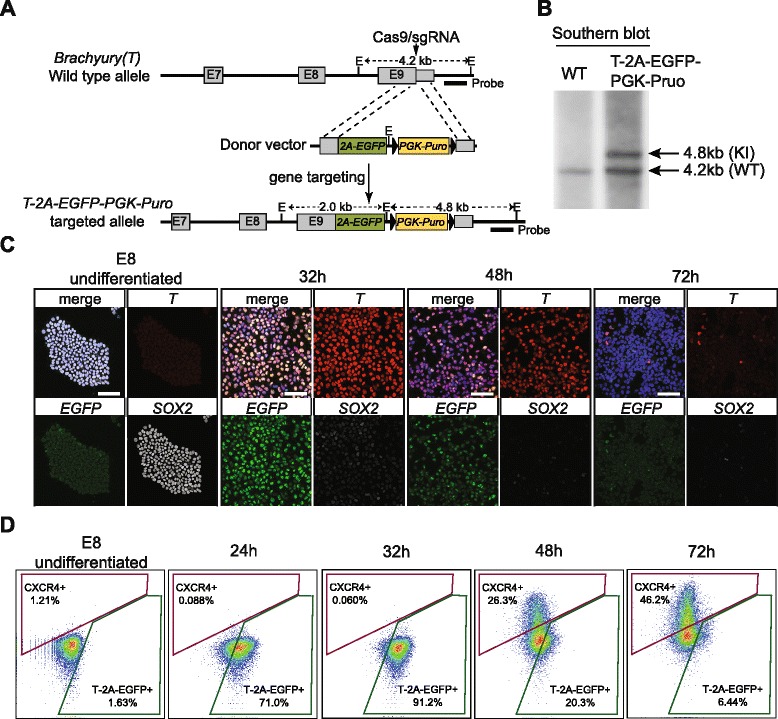
Generation of *T-2A-EGFP* knock-in reporter cell line. **a** Gene targeting strategy of knock-in *T-2A-EGFP-PGK-Puro* cassette to replace the endogenous *T* stop codon, facilitated by the CRISPR/Cas9. *Gray boxes* indicate the exons of the endogenous gene. The arrow indicates the position of the Cas9/sgRNA cut site. The position of the probe for southern blot is indicated. *E EcoRI* sites. The lengths for each EcoRI-digested genomic DNA fragments are indicated. **b** Southern blot shows the targeted allele shifted from 4.2 (wild type genomic fragment, WT) to 4.8 kb (knock-in genomic fragment, KI). **c** Confocal images of SOX2, T, and EGFP immunofluorescence staining over three days of differentiation. Hours of differentiation are indicated. Scale bars = 50 μm. **d** FACS analysis monitoring the dynamics of the percentage of CXCR4^+^ and T-2A-EGFP^+^ cells over three days of differentiation. The *x-axis* indicates GFP/FITC channel. The *y-axis* indicates APC channel

In order to measure the levels of real-time endogenous *T* protein expression, we inserted a *2A-EGFP-PGK-Puro* cassette into the endogenous *T* locus via CRISPR/Cas9-mediated gene targeting (Fig. 4a) [55, 56]. Collective analyses from copy number qPCR, junction PCR, and southern blotting confirmed that clone 39 was a correctly targeted clone with only one copy of the *EGFP* and *Puro* cassette knock-in into the endogenous *T* locus. (Fig. 4b and Additional file 1: Figure S6). Upon removal of the *PGK-Puro* cassette with transient Cre expression, this *T-2A-EGFP* line (clone 39) was used in all the subsequent experiments. Cytogenetic test also verified a normal karyotype after gene targeting and clonal expansion (Additional file 1: Figure S6). Importantly, qPCR analysis on EGFP-sorted cells and immunofluorescence staining confirmed that the dynamics of EGFP expression is highly correlated with both the endogenous *T* transcript and protein expression, respectively, making the *T-2A-EGFP* reporter a robust readout for *T*^*+*^ state in the course of our DE differentiation (Fig. 4c and Additional file 1: Figure S6).

Simultaneously measuring both *T* and *CXCR4* protein levels by FACS provided both the precision and resolution required to detect cell state transitions. After just 24 h of differentiation, more than 70 % of the cells became T-2A-EGFP^+^. The percentage of T-2A-EGFP^+^ cells reached a zenith (approximately 90 %) between 28 and 32 h of differentiation and then gradually decreased over time (20 % at 48 h and below 10 % at 72 h of differentiation, Fig. 4d). Co-staining cells with anti-CXCR4 antibodies revealed that less than 1 % of the cells were CXCR4^+^ at 24 h of differentiation. After 48 h, approximately 25 % of the cells became CXCR4^+^, increasing to above 40 % after 72 h of differentiation (Fig. 4d). Therefore, while T expression transiently peaked around 32 h, CXCR4 expression continually increased, starting as early as 36–40 h of differentiation, a dynamic expression pattern mirroring the temporal profiling by scRNA-seq described above (Fig. 3c). Thus, monitoring the expression of *T-2A-EGFP* and CXCR4 over time presented a tractable means to screen candidate genes identified in the Wave-Crest analysis.

### *KLF8* mediates the mesendoderm to DE transition

**Fig. 5:**
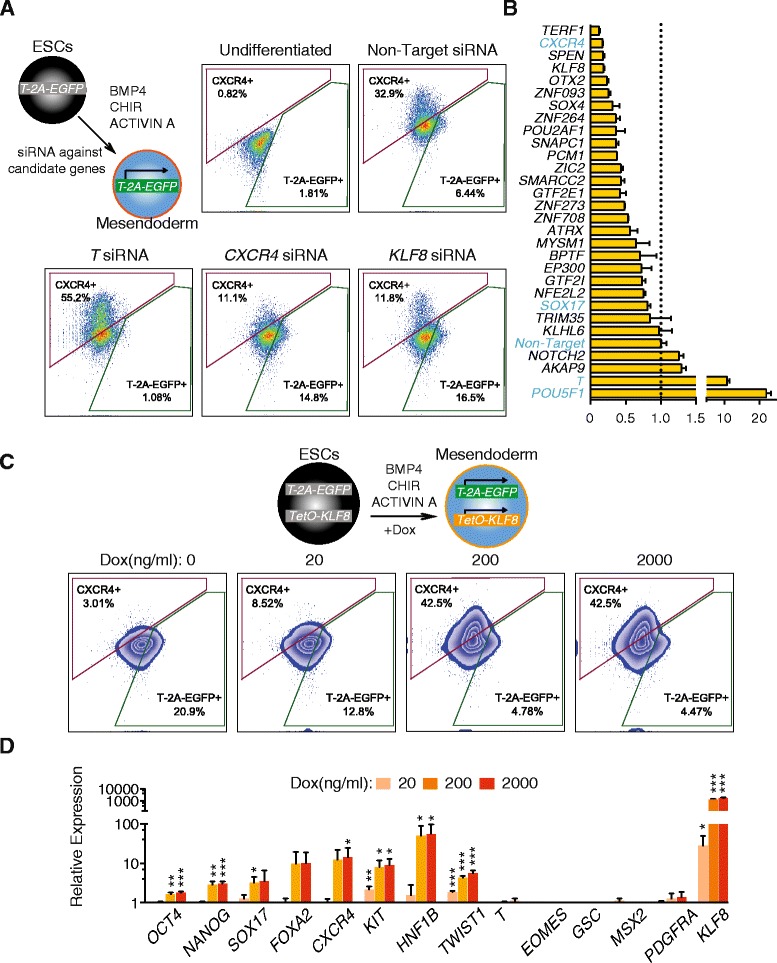
*KLF8* mediates mesendoderm to DE differentiation. **a** Schematics of siRNA knockdown strategy. FACS analysis of CXCR4 and T-2A-EGFP expression at day two of differentiation with representative gene-specific siRNA transfection results. **b** Summary Differentiation Scores of siRNA knockdown experiments of the top 25 genes tested. The *dashed line* indicates a Differentiation Score of 1, set by the non-target siRNA control. Genes in *blue font* indicate control experiments. **c** *Upper panel*, schematics of *KLF8* overexpression strategy during differentiation. *Lower panel*, FACS analysis shows CXCR4 and T-2A-EGFP expression at day two of differentiation. *DOX* Doxycycline. **d** QPCR analysis of three independent *KLF8* overexpression clones tested at day two of differentiation. All data are shown as mean ± S.D. ***p <0.001; **p <0.01; *p <0.05, all by one-tailed t-test. In all FACS plots, the *x-axis* indicates GFP/FITC channel, the *y-axis* indicates APC channel

We hypothesized that if a regulator plays a promotional role during the transition from mesendoderm toward DE, then knocking down its expression would delay this progression. On the other hand, overexpression of such regulators should accelerate this progression. We conducted a siRNA knockdown screen focused on the top 25 upregulated genes identified by Wave-Crest (Fig. 3d). We accounted for both the percentages of EGFP^+^ and CXCR4^*+*^ of each gene knockdown at day two of differentiation (between 45 and 48 h), defined as a “Differentiation Score” for each gene tested (see “Methods”). When compared with non-target siRNA controls (Differentiation Score arbitrarily set to 1), knockdown of *CXCR4* reduced the percentage of CXCR4^+^ cells from greater than 30 % to approximately 10 %; whereas knockdown of *T* substantially reduced the percentage of EGFP^+^ cells and increased percentage of CXCR4^+^ cells, validating the efficacy of siRNA knockdown protocols (Fig. 5a). Among all the genes tested, *TERF1*, *SPEN*, and *KLF8* knockdowns displayed the lowest Differentiation Score (i.e. the most potent blockade of differentiation) (Fig. 5b). Between the top three genes, *KLF8* knockdown showed the smallest increase of T-2A-EGFP expression, indicating a more specific blockade of DE differentiation rather than enhancing mesendoderm or mesoderm differentiation (Fig. 5a and Additional file 1: Figure S7). *KLF8* is expressed at a much lower level in undifferentiated human ES cells than *TERF1* or *SPEN* (Additional file 1: Figure S7). *KLF8* was also detected as a differentially expressed gene in our SCPattern analysis (Additional file 4: Table S3). Collectively, these data support the idea that *KLF8* may play a specific role during the transition from mesendoderm toward DE cells.

**Fig. 6:**
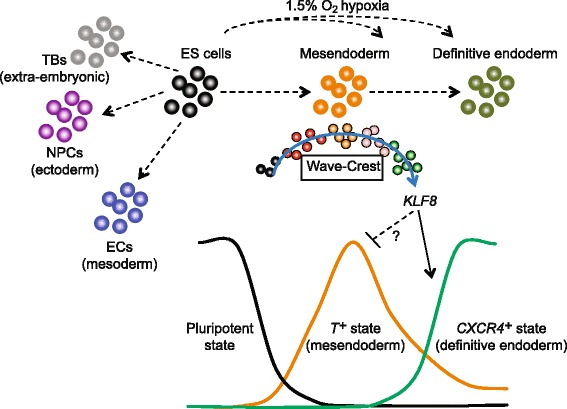
Snapshot and temporal scRNA-seq profiling on progenitor cell states. scRNA-seq from snapshots of lineage-specific progenitors revealed that hypoxia enhances DE differentiation in a time-sensitive manner, likely acting on the mesendodermal progenitors. The differentiation trajectory was reconstructed by Wave-Crest (*blue arrow*) using time course scRNA-seq along the differentiation towards DE cells. Results from loss-of-function and gain-of-function experiments demonstrated that *KLF8* function as a positive regulator mediating cell states transitions from *T* ^*+*^ mesendoderm to *CXCR4* ^*+*^ DE cells. We hypothesize that *KLF8* can suppress *T* ^*+*^ state and in turn enhance the activation of *CXCR4* ^*+*^ state. The molecular mechanisms of how *KLF8* directly or indirectly suppress *T* ^*+*^ state require future investigation

To further examine this possibility, we generated a doxycycline (DOX)-inducible *KLF8* transgene integrated by the PiggyBac system [57] into the *T-2A-EGFP* reporter cell line. Upon differentiation, induction of *KLF8* (DOX treatment from 20 to 40 h of differentiation) robustly increased the percentage of CXCR4^+^ cells from ~5 % to above 40 % in a dosage-dependent manner (Fig. 5c), accompanied by the loss of T-2A-EGFP^+^ cells (Fig. 5c). This result was confirmed by examining a total of three independent *KLF8* overexpression clones. Furthermore, DOX induction of *KLF8* led to significant activation of DE markers including *CXCR4*, *HNF1B*, *SOX17*, and *KIT*, as judged by qPCR analysis from all three clones (Fig. 5d). Importantly, *KLF8* overexpression did not induce the expression of mesendoderm or mesoderm markers such as *T*, *EOMES*, *GSC*, *MSX2*, and *PDGFRA* (Fig. 5d). We also observed that overexpressing *KLF8* increased cell mobility as evident by the upregulation of *TWIST1*, an epithelial-to-mesenchymal transition (EMT) marker (Fig. 5d). These results indicate that *KLF8* plays a specific role promoting the transition from *T*^*+*^ mesendoderm to *CXCR4*^*+*^ DE fate, perhaps through the suppression of *T* or by enhancing the EMT during DE differentiation. These results also suggest that the cell state transition from mesendoderm to DE is a complex and dynamic process, coupling the expression of specific transcriptional regulators with changes in cell migratory behavior. Altogether, our single-cell analysis identified *KLF8* as a previously unrecognized positive regulator of the transition from mesendoderm to nascent DE (Fig. 6). Other genes identified by the single-cell analysis are additional candidates for being regulators of this transition as well. We anticipate that the strategy used in this study – using scRNA-seq analysis to form hypotheses that can be tested by more conventional techniques – may be further applied to uncover novel regulators in other lineages during cell fate decisions both in vitro and in vivo.

## Discussion

### scRNA-seq from lineage progenitors and the role of hypoxia on DE differentiation

Our scRNA-seq analysis of distinct progenitors derived from human ES cells revealed that one of the principal components (PC5) discriminated the DE cells from all other progenitors (Fig. 1d). Allez enrichment analysis on the gene loadings from PC5 revealed a role for metabolism in the genesis of DE cells (Figs. 1e and 2). Among the human ES cells tested, culture conditions of 1.5 % O_2_, but not 5 % O_2_, consistently increased the percentage of CXCR4^+^ cells (Fig. 2b). Most importantly, the impact of hypoxia is pronounced in the first two days of differentiation (Fig. 2d and e). Interestingly, only one recent report thus far described a positive effect of hypoxia on differentiating mouse ES cells to DE [43]. However, given that mouse and human ES cells are maintained by distinct signaling pathways [58, 59], it was not entirely clear if the hypoxia-enhanced DE differentiation may or may not directly translate to human ES system. To the best of our knowledge, our study is the first to investigate the effects of hypoxia during early definitive endoderm differentiation from human ES cells.

Most importantly, the implications from these results are twofold. First, there is a critical time window when the precursors of DE are responsive to hypoxic conditions, causing the initial upregulation of DE markers (Fig. 6). Second, it is plausible that cells destined to become DE cells, experience a lower oxygen concentration that prompts their specification to DE fate. Future whole embryo culture experiments will determine if a hypoxic microenvironment can enhance DE formation/specification.

### Temporal scRNA-seq analysis highlights the transcriptomic transitions from pluripotent to mesendoderm to DE states

We compared scRNA-seq profiles between each pair of neighboring time points over four days of differentiation (Fig. 3a) and found that the number of differentially expressed genes generally decreased over time (2224 genes [0–12 h], 830 genes [12–24 h], 1317 genes [24–36 h], 466 genes [36–72 h], and 11 genes (72-96 h)]. This observation suggests that transcriptional changes gradually decrease with continual BMP4 and Activin A signaling. Interestingly, our PCA plot (Fig. 3a) shows overlapped individual cells collected between 72 h and 96 h, suggesting that cells could gradually transition into a relatively “stable” state at 72 h of differentiation.

When focusing on the 36 h time point of differentiation, the reconstructed cell order provides a glimpse of how the cells progress over time (Fig. 3c and Additional file 1: Figure S5). This analysis reveals that DE markers *SOX17* and *CXCR4* are activated surprisingly early in a small number of cells with low or no expression of *T* (Fig. 3c and Figure S5). This trend, consistent with previous findings, confirmed the reconstructed trajectory from mesendoderm toward DE and provided a measure of confidence in the subsequent fishing step. While we only tested upregulated genes from the Wave-Crest analysis, it is possible that our top downregulated genes are playing regulatory roles for mesendoderm or mesoderm fate differentiation (Fig. 3d). Given the high quantity of reconstructed single-cell information, follow-up experiments are needed to investigate the full set of regulators governing the decision to bifurcate from mesendoderm to either mesoderm or DE.

It is not surprising that scRNA-seq data revealed cell state asynchrony during differentiation as the phenomenon has been reported in other cell types subjected to scRNA-seq analysis [26–28]. However, what factors contribute to the cell-to-cell delay or advancement of transcriptomic progression is still unclear. One possible factor is the cell cycle, which is unsynchronized across the population of cells [25] either at the initiation of differentiation and/or when single cells were collected for RNA-seq. For example, recent reports suggest that the cell cycle status can influence signaling pathways directing exit from the pluripotent state [60–62]. To resolve this, future efforts will need to monitor real-time expression of lineage specification markers while simultaneously tracing cell division.

Two new statistical tools to characterize our temporal scRNA-seq data are reported in this study. The first is SCPattern, an empirical Bayes model to identify genes with expression changes over time, specifically designed to tackle the analytical challenges of scRNA-seq data. The statistical power and simulations of SCPattern are detailed in a companion study [29]. The second tool, Wave-Crest, is composed of a first step to reconstruct temporal cell order and a second step focused on identifying novel regulators (fishing) (see “Methods”). Evaluating the performance of Wave-Crest by simulation studies can be found in Additional file 1: Supplementary Results and Figure S8 with Additional file 7. Wave-Crest attempts to reorder cells along their differentiation progression based on known markers expression and focused on identifying novel regulators at transition state (particularly from mesendoderm to DE). Wave-Crest also uses known temporal information to improve its performance. While the development of Wave-Crest was not intended to perform pseudo-temporal reconstruction, its reconstructed single-cell order could represent a particular differentiation trajectory guided by preselected markers. While comparing the performance between Wave-Crest and other pseudo-temporal approaches is beyond the scope of this study, a preliminary comparison of using known markers to guide single-cell reordering between Wave-Crest and Monocle [63] could be found in Additional file 1: Supplementary Results and Figure S9. It is important to note that this comparison only used our preselected markers. A graphical user interface implementation of Wave-Crest is also available; details may be found in Additional file 1: Supplementary Methods and Figure S10. To decide candidate genes to use for reordering, the algorithm currently utilizes differential expression results from SCPattern combined with prior knowledge of markers (Additional file 1: Figure S5). Although supervised or semi-supervised learning plays a role in most of the single-cell analyses [27, 64–66], it would be ideal to extract markers from high dimensional data in an unsupervised fashion. The fishing step is performed primarily for transcriptional regulators in this study. It will be of great interest to perform fishing for other categories of gene families, such as post-translational regulators or regulatory RNA species shown to be critical for cell fate transitions. Finally, control of cell-to-cell variabilities by spike-ins was not applied in our scRNA-seq data due to technical challenges in our initial in-house trial experiments. Future efforts to employ novel computational approaches to access scRNA-seq variability should greatly improve our ability to identify additional novel regulators [30, 32, 50, 66–68].

### *T-2A-EGFP* reporter line as a robust platform to define the role of *KLF8* in mediating DE differentiation

In order to directly test the candidate genes identified from scRNA-seq analysis, we generated the first *T*-knock-in reporter human ES cell line that faithfully reflects the endogenous *T* protein expression level, rather than at the transcriptional level [69, 70]. As *T* activation marks the onset of gastrulation, this reporter line will be useful for future studies involving the earliest molecular events during the formation of the primitive streak state in human pluripotent stem cells. While CXCR4 antibody staining provided a robust readout for DE state, combining our *T-2A-EGFP* reporter with other genetically tagged reporters of transcription factor whose expression is important for DE cell fate would be beneficial.

Our siRNA screen resulted in mostly delayed differentiation phenotypes among the candidate genes (top upregulated genes) tested (Fig. 5b), consistent with the hypothesis that this group of genes is mostly positive regulators of the DE fate. Specifically, *TERF1*, *SPEN*, and *KLF8* stand out as the strongest candidates to reduce the percentage of *CXCR4*^*+*^ cells (Fig. 5b and Additional file 1: Figure S7). We concentrated on *KLF8* because it displayed a dynamic expression pattern over the course of four days of differentiation (Additional file 1: Figure S7). The knockdown of *TERF1* and *SPEN* are likely reflecting a pleiotropic rather than specific effect on DE differentiation. Interestingly, our data also indicate that *KLF8* could negatively regulate *T* expression since *KLF8* knockdown increased *T* expression (Fig. 5a) and *KLF8* overexpression decreased *T* expression (Fig. 5c). Whether *T* is a direct target of *KLF8* requires further examination (Fig. 6). The positive regulation role of *KLF8* in DE differentiation is best demonstrated by overexpression of *KLF8* alone (which is sufficient to induce DE markers) suggesting an acceleration of the transition from mesendoderm to DE (Figs. 5d and 6).

While the exact role(s) of *KLF8* during early embryogenesis has not been examined in detail, a hemizygous gene-trapped *KLF8* allele exhibits developmental delay at mid-gestation; although with variable penetrance in the gene-trapped embryos examined [71]. Furthermore, recent studies indicate that *KLF8* mediates a number of oncogenic processes including transformation and metastasis in ovarian, breast, bladder, and colorectal cancers [72–74]. It is plausible that *KLF8* may regulate the EMT that is coupled with DE specification (Fig. 6). Future efforts may elucidate the molecular mechanisms by examining the direct targets of *KLF8* during DE cell fate specification*.*

## Conclusions

In this study, we reported the analysis of scRNA-seq data from a total of 1776 single cells generated from various lineage-specific progenitors and from time course profiling along mesendoderm toward DE lineage. To our knowledge, this is one of the most complete scRNA-seq studies characterizing human ES cells and their progenies to date. We describe new algorithms for scRNA-seq analysis: Wave-Crest and SCPattern. Our scRNA-seq analyses uncovered a cohort of novel regulators potentially responsible for the transitioning phase from mesendoderm toward endodermal progenitors. We have demonstrated that *KLF8* plays a pivotal role in accelerating the differentiation of DE cells. Altogether, we believe that the combination of scRNA-seq analysis and genetic approaches will shed light on novel molecular mechanisms governing cell fate decisions.

## Methods

### Cell culture and differentiation

H1 and H9 human ES cells were routinely maintained at the undifferentiated state in E8 medium on Matrigel (BD Bioscience) coated tissue culture plates with daily medium feeding [34]. Human ES cells were passaged every 3–4 days with 0.5 mM EDTA in PBS at 1:10 to 1:15 ratio for maintenance. H1 were differentiated according to previously established protocols [6, 17, 35]. In brief, H1 cells were individualized with Accutase (Life Technologies), washed once, and then seeded onto Matrigel coated plates at a density of 1–2.0 × 10^4^ cells/cm^2^ and cultured in various differentiation medium. For TBs, H1 were seeded in E7 (E8 minus FGF2) with 50 ng/mL BMP4 and SB431542 (5 μM). For ECs, H1 were seeded in E8 with 5 ng/mL BMP4 and 25 ng/mL Activin A for the first two days, then switched to supplement with VEGF and WNT for three days. For DE cells, H1 cells were seeded in E8 with BMP4 (5 ng/mL), Activin A (25 ng/mL), and CHIR99021 (1 μM) for the first two days, then withdraw CHIR99021 for the remaining period of differentiation. This differentiation protocol is used in all RNA-seq, hypoxic, siRNA knockdown and *KLF8* overexpression experiments. For NPCs, the undifferentiated *H1-SOX2-mCherry* reporter line was treated with 0.5 mM EDTA in PBS for 3–5 min and seeded in NPC differentiation medium (1–2 × 10^4^ cells/cm^2^). The NPC differentiation medium consists of E6 (E8 minus FGF2, minus TGFβ1), with 2.5 μg/mL insulin, SB431542 (10 μM) and 100 ng/mL Noggin [35]. DE cells, ECs, and TBs were harvested at the end of day 5 for antibody staining and subsequent FACS sorting. Specifically, ECs were enriched from the PECAM1^+^/CD34^+^ double positive sorted population; DE cells were enriched from the CXCR4^+^ sorted population; TBs were enriched from the TROP2^+^ sorted population; and NPCs were enriched from sorting for the Cherry + population from a SOX2-Cherry^+^ knock-in line at the end of day 7 of differentiation. For the time course scRNA-seq experiments, no cell sorting or marker enrichment was performed prior to capture single cells. All differentiation media were changed daily. HFFs were cultured in DMEM/F12 supplemented with 10 % FBS. All 1.5 % O_2_ hypoxia experiments were conducted in a hypoxic glove box (Coy Labs). All 5 % O_2_ hypoxia experiments or normoxia 20 % O_2_ are conducted in standard cell culture incubators with [N_2_] regulators. All the cell cultures performed in our laboratory have been routinely tested as negative for mycoplasma contamination and authenticated by cytogenetic tests.

### Single-cell capture and single-cell cDNA library preparation

Single-cell loading, capture, and library preparations were performed using the Fluidigm C1 system as described previously [25]. 5000–8000 cells were loaded onto a medium-sized (10–17 μm) C1 Single-Cell Auto Prep IFC (Fluidigm) and the cell-loading script was used according to the manufacturer’s instructions. The capture efficiency was inspected using EVOS FL Auto Cell Imaging system (Life Technologies) to perform an automated area scanning of the 96 capture sites on the IFC. Empty capture sites or sites having more than one cell captured were first noted, and those samples were later excluded from further library processing for RNA-seq. Immediately after capture and imaging, reverse transcription and cDNA amplification were performed in the C1 system using the SMARTer PCR cDNA Synthesis Kit (Clontech) and the Advantage 2 PCR Kit (Clontech) according to the instructions in the Fluidigm user manual. Full-length, single-cell cDNA libraries were harvested the next day from the C1 chip and diluted to a range of 0.1–0.3 ng/μL. Diluted single-cell cDNA libraries were fragmented and amplified using the Nextera XT DNA Sample Preparation Kit and the Nextera XT DNA Sample Preparation Index Kit (Illumina). Libraries were multiplexed at 24 or 48 libraries per lane, and single-end reads of 67 bp were sequenced on an Illumina HiSeq 2500 system.

### Immunofluorescence staining and confocal image analysis

Cells were seeded and cultured on Matrigel coated glass-bottom culture dishes (MatTek, 12- or 24-well dishes) for differentiation or treatments. Cultured cells were then washed with PBS and with BD Perm/wash buffer and then fixed with BD Cytofix at 4 °C for 15 min. Cells were then permeablized with 0.2 % TritonX-100 (in PBS) at room temperature for 30 min. Cells were then blocked with blocking buffer (2 % BSA and 1 % FBS in PBS) for 1 h at room temperature followed by staining with primary antibody diluted in blocking buffer at 4 °C overnight. The next day, cells were washed three times with blocking buffer before incubated with AlexaFluor secondary antibodies (Invitrogen, 1:1000 dilutions in blocking buffer) and DAPI for 1 h at room temperature. Cells were then washed three times with blocking buffer and mounted on glass slides (Vectors Labs) for imaging. All the primary antibodies used in this study can be found in Additional file 8: Table S7. Immunofluorescence images were collected using a Nikon A1R laser scanning confocal microscope with Plan Apo 10x, Plan Fluor 20x Ph1 DLL, or Plan Apo 20x DIC M objectives. Images were processed using NIS Elements or ImageJ. Some z-stacks were presented as maximum intensity projection images.

### Gene targeting and plasmids construction

#### Brachyury (T)-2A-EGFP reporter

*T-2A-EGFP-PGK-Puro* targeting vector (donor vector) with ~480 bp homology arms on each side of the stop codon was cloned into the targeting vector backbone (modified from Addgene 31938) [75]. FseI and SacI linearized *T-2A-EGFP-PGK-Puro* cassettes were used for gene targeting experiments. All the DNA oligos used in this study are listed in Additional file 9: Table S8. CRISPR/Cas9 mediated gene targeting experiments were performed as previously described [55]. Briefly, H9 cells were individualized with Accutase, washed once with E8 medium, and resuspended in E8 medium with 10 mM HEPES buffer (pH 7.2–7.5) (Life Technologies). For electroporation, 2.5 × 10^6^ cell were mixed with 7.5 μg of *pCMV-hCas9* plasmid (Addgene 41815) [56], 7.5 μg of sgRNA construct, and 10 μg of linearized donor DNA template in a total of 500 μL of cell/DNA suspension, transferred to a 4-mm cuvette (Bio-Rad) and electroporated with a Bio-Rad Gene Pulser Xcell. Electroporation parameters were 250 V, 500 μF, and infinite resistance. Cells were then plated into Matrigel-coated culture dishes in E8 medium supplied with 10 μM ROCK inhibitor Y-27632 (Tocris). Medium was changed daily. Puromycin selection was started three to four days after electroporation. Puromycin-resistant colonies were picked five to seven days after drug selection was applied. In the *T-2A-EGFP* targeting experiments, clone 39 was verified as correctly targeted clone with normal karyotype and is used for all the subsequent experiments in this study (Additional file 1: Figure S6). For the SOX2-Cherry reporter, a *Cherry-2A-Puro* cassette (donor vector) with ~480 bp homology arms on each side of the stop codon was cloned into the targeting vector backbone (modified from Addgene 31939) [75]. FseI and SacI linearized *SOX2-Cherry-2A-Puro* cassettes were used for gene targeting performed in the same manner as described above. Corrected targeted clones were confirmed by PCR and southern blot analysis. Details about the *SOX2* gene targeting will be reported elsewhere (Chu et al., in preparation). All vectors and their sequences are available upon request.

### The Wave-Crest method

Wave-Crest extends the nearest-insertion algorithm to recover single-cell order following the expression of preselected markers in time course scRNA-seq data. Prior to the analyses, we scaled expression within each gene to values with mean 0 and variance 1 to ensure the values across different genes are comparable. Wave-Crest reorders cells within each time point by utilizing information from its other time points. Similar to Leng et al. [25], the Wave-Crest algorithm implements an extended nearest insertion (ENI) algorithm to reorder the cells, but with a constraint that cells from different collection time are not allowed to be mixed in the recovered order. The Wave-Crest ENI starts with *τ* randomly selected cells, one from each time point. These *τ* cells are sorted by the time course order. A *τ* + 1 th cell is chosen at random and inserted into the series of cells. Suppose that this cell is from time point *t*_1_, this forms two candidate orders – insert this cell between the *t*_1_ − 1 th and *t*_1_ th cell in the original order, or between *t*_1_ th and *t*_1_ + 1 th. We evaluate each order using the aggregated mean squared error (MSE) of a polynomial regression. For a given order, the polynomial regression is fitted to the rescaled expression of each gene. For each order, the aggregated MSE of a candidate gene group is defined as the summation of the MSEs among all genes of interest. The optimal order of the first *τ* + 1 cells is then selected as the one that minimizes the aggregated MSE. This process is repeated to insert the *τ* + 2 th cell and so on, until all cells are considered. A 2-opt algorithm is then applied to avoid finding local maxima and to refine the global expression profile [54]. Evaluation of the ENI algorithm and the 2-opt algorithm may be found in Additional file 1: Supplementary Results and Figure S8e. Wave-Crest also incorporates a detection step to further identify genes with a smooth profile following the reconstructed pseudo-time, which we called “fishing”. This detection (fishing) step again utilizes polynomial regression. For a gene g, we reorder its rescaled expression following the recovered order, fit a polynomial curve, and calculate its gene-specific MSE (observed MSE). A permutation test is then conducted to determine the goodness of the fit by comparing the observed MSE to a large group of simulated genes. To generate a permuted gene, we randomly pick a gene from the full set of genes under consideration and permute its cell order, fit a polynomial regression as above and calculate the MSE of the permuted gene. The MSE distribution of the permuted genes is then used to make inference about the MSE distribution under the null hypothesis (no expression change associated with the reconstructed cell order). An empirical gene’s permutation *p* value is then calculated as $\frac{\#\kern0.1em \mathrm{permuted}\kern0.3em \mathrm{M}\mathrm{S}\mathrm{E}\le \kern0.3em \mathrm{observed}\kern0.3em \mathrm{M}\mathrm{S}\mathrm{E}}{\#\kern0.1em \mathrm{permuted}\kern0.3em \mathrm{M}\mathrm{S}\mathrm{E}}$, where the observed MSE indicates the g’s MSE based on the reconstructed order. Genes with small permutation *p* values are considered detected. Wave-Crest is available as an R package, freely available at (https://github.com/lengning/WaveCrest). The softwares are licensed under the terms of the Apache License 2.0. A graphical user interface is also provided (Additional file 1: Figure S10), which allows users with little computational background to perform the analysis.

In the DE differentiation time course analysis described in this manuscript, the ENI algorithm and 2-opt algorithm were applied on 758 cells across six time points (0 h, 12 h, 24 h, 36 h, 72 h, and 96 h). Genes whose median expression is less than 10 were omitted. The fishing step was applied on the 172 cells collected at 36 h of differentiation (following the ENI reconstructed cell order). A total of 2178 transcriptional regulators were considered in this fishing step (Additional file 5: Table S4). We defined the top genes with expression trend within 36 h by taking the ones with small MSE in the polynomial fitting along these 172 cells. The genes were further classified into upregulated and downregulated groups by their expression trend along the recovered order of these 172 cells. The two groups were defined by the sign of the slope coefficient in gene-specific linear fitting. The genes with positive (negative) slope coefficient were defined as up- (down-) regulated from early-36 h cells to late-36 h cells. A total of 100,000 permutations were conducted in the permutation test.

### Reverse transcription (RT) and qPCR analysis

All procedures were performed as described previously [76]. Total RNAs were purified using RNeasy kits (Qiagen) with either on-column DNase treatment or genomic DNA removal columns. Between 100 ng to 500 ng of purified RNAs were reverse transcribed with SuperScript VILO Master mix (Life Technologies). To perform TaqMan qPCR reactions (10 μL total volumes), 1 μL of the cDNA was subsequently used in each of the triplicate qPCR reactions with individual 1× TaqMan Gene Expression assays and 1× TaqMan Universal PCR Master Mix II (Life Technologies). qPCR was performed using ViiA™ 7 System and data analysis was performed using ExpressionSuite™ (all from Life Technologies). All TaqMan Gene Expression assays are from Life Technologies and are listed in Additional file 9: Table S8.

### siRNA knockdown experiments

All the siRNA are from ON-TARGET*plus* siRNA SMARTpool (Healthcare/Dharmacon), listed in Additional file 10: Table S9. siRNA were dissolved in 1 × siRNA buffer and stored as 16 μM stock in –80 °C. One hour after cells were plated for differentiation, a final concentration of 50 nM of each siRNA pool was transfected with RNAi MAX with OPTIM-DMEM following manufacture’s protocols (Life Technologies). Each gene knockdown was performed with at least two replicates of experiments. FACS analysis was performed to measure the percentages of CXCR4^+^ and EGFP^+^ cells for each gene knockdown at 42–48 h post differentiation. The Differentiation Score is calculated as the (% of CXCR4^+^ cells)/(% of T-2A-EGFP^+^ cells). The score obtained from non-targeting control siRNA (as transfection control, arbitrarily set to 1), was used to normalize all the results obtained from individual gene-specific siRNA transfection experiments.

### PiggyBac vector construction and *KLF8* overexpression clones

The cDNA of *KLF8* was obtained from GeneCopoeia as Gateway Entry vectors (GC-T1091), and was subsequently cloned into a PiggyBac Gateway Destination vector using Gateway LR clonase (Life Technologies), placing *KLF8* downstream of a DOX-inducible promoter (*pB-TetO-KLF8*) as previously reported [57]. A separate PiggyBac vector encoding *pEF1a-rtTA-IRES-Puro* cassette (DOX-responsive transactivator along with puromycin resistance gene) was co-electroporated with *pB-TetO-KLF8* and *CMV-hyPBase* plasmids or *hyPBase* mRNA. For electroporation, 2.5 × 10^6^ of *H9-T-2A-EGFP* reporter cells were mixed with 30.0 μg of *pB-TetO-KLF8* plasmid, 1.5 μg of *pEF1a-rtTA-IRES-Puro* plasmid, and 1.5 μg of *CMV-hyPBase* in a total of 500 μL of cell/DNA suspension, transferred to a 4-mm cuvette (Bio-Rad), and electroporated with a Bio-Rad Gene Pulser Xcell. Electroporation parameters were 250 V, 500 μF, and infinite resistance. Cells were then plated into Matrigel-coated culture dishes in E8 medium supplied with 10 μM ROCK inhibitor Y-27632 (Tocris). Medium was changed daily. Puromycin selection (1.0 μg/mL) was applied three days after electroporation. Puromycin-resistant colonies were picked approximately seven days after drug selection was applied. Three independent clones with the most uniformed expression of *KLF8* upon DOX treatment (2.0 μg/mL) were selected for subsequent experiments. The vectors and their sequences are available upon request.

### Bulk RNA-seq library construction

Bulk RNA samples were collected at the time when the single-cell samples were processed. Cell pellets were lysed in Buffer RLT (Qiagen) and stored in –80 °C until RNA isolations. Total RNA of each cell type was purified using the RNeasy Kit (Qiagen). cDNA libraries were prepared and indexed with Illumina’s TruSeq RNA Sample Prep Kit v2 and sequenced on Illumina’s HiSeq 2500 system with 4–6 indexed samples per lane with 51 bp single-end reads.

### Read processing and mapping

Reads were mapped via Bowtie 0.12.8 [77] against the hg19 RefSeq reference (“NM_” designated genes and mitochondrial genes from the Illumina iGenomes annotation). The mapping allows up to two mismatches and up to 20 multiple hits. The expected counts and TPMs were quantified via RSEM 1.2.3 [78].

### Bulk data – snapshot of progenitor cell types

A total of 19 bulk samples from seven cell types were sequenced: duplicates for DEC, NPC, and TB; triplicates for H9, EC, and HFF; and four replicates for H1. The bulk RNA-seq data were normalized by median-by-ratio normalization.

### scRNA-seq data – snapshot of progenitor cell types

In total, seven cell types were considered. Cells having fewer than 5000 genes with TPM >1 were removed in quality control. A total of 1018 cells passed the quality control. In more detail, 212, 162, 138, 105, 159, 173, and 69 cells were considered in H1, H9, DEC, EC, HFF, NPC, and TB, respectively. The scRNA-seq data were normalized by median-by-ratio normalization. Genes with potential ordering effect (OE) were removed prior to downstream analyses. The OE genes were detected using OEFinder [79]. A gene is called OE if it has an OEFinder *p* value ≤0.01 in at least one cell type. A total of 392 OE genes were removed in various cell type datasets.

### Bulk data – time course experiment

Triplicates were sequenced from each of the six time points – 0 h, 12 h, 24 h, 36 h, 72 h, and 96 h. The bulk RNA-seq data were normalized by median-by-ratio normalization.

### scRNA-seq data – time course experiment

In total, six time points were considered. Cells having fewer than 5000 genes with TPM >1 were removed in quality control. A total of 758 cells passed the quality control. In more detail, 92, 102, 66, 172, 138, and 188 cells were considered in 0 h, 12 h, 24 h, 36 h, 72 h, and 96 h, respectively. The scRNA-seq data were normalized by median-by-ratio normalization. Genes with potential OE were removed prior to downstream analyses. A total of 536 OE genes were removed in the time course dataset.

### Bulk-supervised PCA

Previous publications showed that directly performing PCA on single-cell data may capture unwanted noise [80]. Therefore, bulk-supervised PCA were conducted to investigate differences between different cell types in the following manner. Denote the normalized bulk RNA-seq expression of gene *g* in sample *s* as *Y*_*g*_^*s*^. Prior to PCA analysis, for each gene *g*, the bulk RNA-seq expression values were rescaled to values with mean 0 and standard deviation 1 (denote as *Ỹ*_*g*_^*s*^). Similarly, denote normalized scRNA-seq expression of gene *g* in cell *j* as *X*_*g*_^*j*^ and denote the rescaled data as ${\tilde{X}}_g^j$. We applied PCA on the rescaled bulk RNA-seq data and obtain loadings of PCs. Denote loading of gene *g* in PC *n* as *W*_*g*_^*n*^. The bulk-supervised PCA transformed data of cell *j* in PC *n* is then calculated as ${\displaystyle {\sum}_g{W}_g^n{\tilde{X}}_g^j}$.

### Differential expression analysis in scRNA-seq time course data by SCPattern

SCPattern was used to identify genes with expression changes in the scRNA-seq time course data. For each gene, SCPattern calculates the posterior probability of being each possible expression path (e.g. Up-Up-Up-Up-Up, Up-Up-Up-Up-Down, etc.). A gene is called “differentially expressed” if its most likely path is not EE-EE-EE-EE-EE (EE, equal expression). A total of 3247 differentially expressed genes were identified in the scRNA-seq time course experiment listed in Additional file 4: Table S3. The development of SCPattern is detailed in a companion study [29]. Both SCPattern and its graphical interface implementation are freely available at https://github.com/lengning/SCPattern. The software is licensed under the terms of the Apache License 2.0.

### Additional software used in this study

To generate figures and text, the following software packages were used: Microsoft Word, Excel, and PowerPoint for Mac v14.5.6; Adobe Illustrator CSS v15.0.2; Endnote X7.3.1; and Prism 6 for Mac v6.0e. Fluidigm SINGuLAR Analysis Toolset (fluidigmSC v.3.0.3).

## Abbreviations

DE, definitive endoderm; DEC, definitive endoderm cell; DOX, doxycycline; EC, endothelial cell; EMT, epithelial-to-mesenchymal transition; ES, embryonic stem; FACS, fluorescence-activated cell sorting; GO, gene ontology; HFF, human foreskin fibroblast; NPC, neuronal progenitor cell; PCA, principal component analysis; scRNA-seq, single-cell RNA sequencing; TB, trophoblast-like cells

## Additional files

Additional file 1: Figure S1. Experimental outline and qualities of scRNA-seq on human ES-derived progenitors. **Figure S2.** PCA and heterogeneity analysis of human ES-derived progenitors. **Figure S3.** Characterizations of the impacts of hypoxia on DE differentiation. **Figure S4.** Quality control and PCA of time course scRNA-seq experiments. **Figure S5.** Genes used for Wave-Crest to reconstruct DE differentiation trajectory. **Figure S6.** Characterizations of *T-2A-EGFP* knock-in reporter. **Figure S7.** Summary of siRNA experiments and characterization of top candidate genes. **Figure S8.** Simulation results evaluating Wave-Crest. **Figure S9.** Monocle results on DE differentiation. **Figure S10.** Screenshot of Wave-Crest graphical user interface. **Supplementary Results** and **Supplementary Methods**. (PDF 2613 kb) Additional file 2: Table S1. List of genes used in Fig. 1c for hierarchical clustering. (XLSX 30 kb) Additional file 3: Table S2. List of the GO terms by Allez enrichment analysis. (XLSX 62 kb) Additional file 4: Table S3. List of SCPattern identified stage-specific 3247 genes. (XLSX 167 kb) Additional file 5: Table S4. List of 2178 genes used for Wave-Crest fishing step. (XLSX 54 kb) Additional file 6: Table S5. List of the top 150 genes identified by Wave-Crest fishing step. (XLSX 56 kb) Additional file 7: Table S6. List of kernel signals for Wave-Crest simulation studies. (XLSX 21 kb) Additional file 8: Table S7. List of the antibodies used in this study. (XLSX 43 kb) Additional file 9: Table S8. List of the DNA oligos and qPCR assays used in this study. (XLSX 32 kb) Additional file 10: Table S9. List of the siRNA used in this study. (XLSX 46 kb)
