# Supplementary material for: Single-cell RNA-seq reveals novel regulators of human embryonic stem cell differentiation to definitive endoderm
Source: Genome Biol. 2016 Aug 17;17(1):173. doi: 10.1186/s13059-016-1033-x (PMC4989499; doi:10.1186/s13059-016-1033-x)
Supplement: Additional file 1: Figure S1. — Experimental outline and qualities of scRNA-seq on human ES-derived progenitors. Figure S2. PCA and heterogeneity analysis of human ES-derived progenitors. Figure S3. Characterizations of the impacts of hypoxia on DE differentiation. Figure S4. Quality control and PCA of time course scRNA-seq experiments. Figure S5. Genes used for Wave-Crest to reconstruct DE differentiation trajectory. Figure S6. Characterizations of T-2A-EGFP knock-in reporter. Figure S7. Summary of siRNA experiments and characterization of top candidate genes. Figure S8. Simulation results evaluating Wave-Crest. Figure S9. Monocle results on DE differentiation. Figure S10. Screenshot of Wave-Crest graphical user interface. Supplementary Results and Supplementary Methods. (PDF 2613 kb) [file 13059_2016_1033_MOESM1_ESM.pdf]

Additional file 1 for

Single-cell RNA-seq reveals novel regulators of human embryonic stem cell differentiation to definitive endoderm

Li-Fang Chu[1*†], Ning Leng[1,6†], Jue Zhang[1], Zhonggang Hou[1, 7], Daniel Mamott[1], David T. Vereide[1], Jeea Choi[4], Christina Kendziorski[5], Ron Stewart[1] and James A. Thomson[1, 2, 3*]

[1]Regenerative Biology, Morgridge Institute for Research, Madison, WI 53715, USA.
[2]Department of Cell & Regenerative Biology, University of Wisconsin-Madison, Madison, WI, USA.
[3]Department of Molecular, Cellular, & Developmental Biology, University of California, Santa Barbara, CA, USA.
[4]Department of Statistics, University of Wisconsin, Madison, WI, USA.
[5]Department of Biostatistics and Medical Informatics, University of Wisconsin, Madison, WI, USA.
[6]Present address: Genentech, Inc., South San Francisco, CA, USA.
[7]Present address: Department of Cell Biology, Harvard Medical School, Boston, MA, USA.
[†]Equal contributors

*Corresponding authors:

James A. Thomson, V.M.D., Ph.D.

Director of Regenerative Biology, The Morgridge Institute for Research.

330 N Orchard St. Madison, WI 53715. phone: 608-316-4346

Email: jthomson@morgridge.org

Li-Fang Chu, Ph.D.

Assistant Scientist, The Morgridge Institute for Research.

330 N Orchard St. Madison, WI 53715. phone: 608-890-4244

Email: lchu@morgridge.org

Listed of Supplementary Figures in Additional file 1:

Figure S1: Experimental outline and qualities of scRNA-seq on human ES-derived progenitors.
Figure S2: PCA and heterogeneity analysis of human ES-derived progenitors.
Figure S3: Characterizations of the impacts of hypoxia on DE differentiation.
Figure S4: Quality control and PCA of time course scRNA-seq experiments.
Figure S5: Genes used for Wave-Crest to reconstruct DE differentiation trajectory.
Figure S6: Characterizations of *T-2A-EGFP* knock-in reporter.
Figure S7: Summary of siRNA experiments and characterization of top candidate genes.
Figure S8: Simulation results evaluating Wave-Crest.
Figure S9: Monocle results on DE differentiation.
Figure S10. Screenshot of Wave-Crest graphical user interface.
Supplementary Results
Supplementary Methods

Figure S1

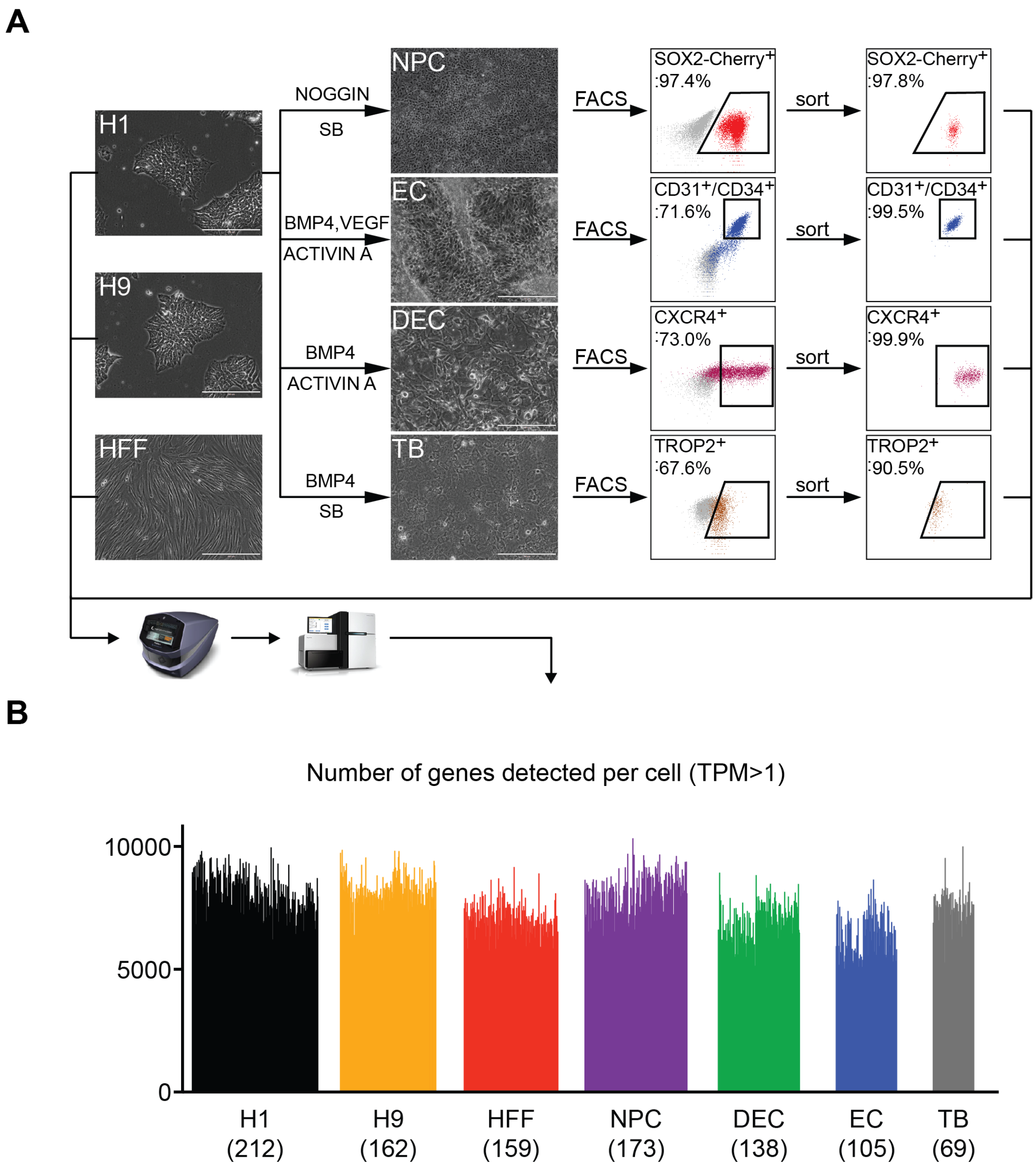

Fig. S1. Experimental outline and qualities of scRNA-seq on human ES-derived progenitors. **a** H1 human ES cells were differentiated to lineage-specific progenitors with various cocktails of growth factors as indicated. Progenitors were FACS sorted with indicated cell surface markers or reporters (also see Methods). Undifferentiated H1 or H9 ES cells and HFFs were collected to serve as controls. All the cell types were captured for scRNA-seq using Fluidigm C1 system. Scale bar = 200 µm. **b** Number of genes detected per cell with TPM > 1. Each individual colored bar represents the data from one cell of indicated cell type. Numbers of single cells profiled for each cell type are indicated in parentheses. NPCs = neuronal progenitor cells. DECs = definitive endoderm cells. ECs = endothelial cells TBs = trophoblast-like cells. HFFs = human foreskin fibroblasts.

Figure S2

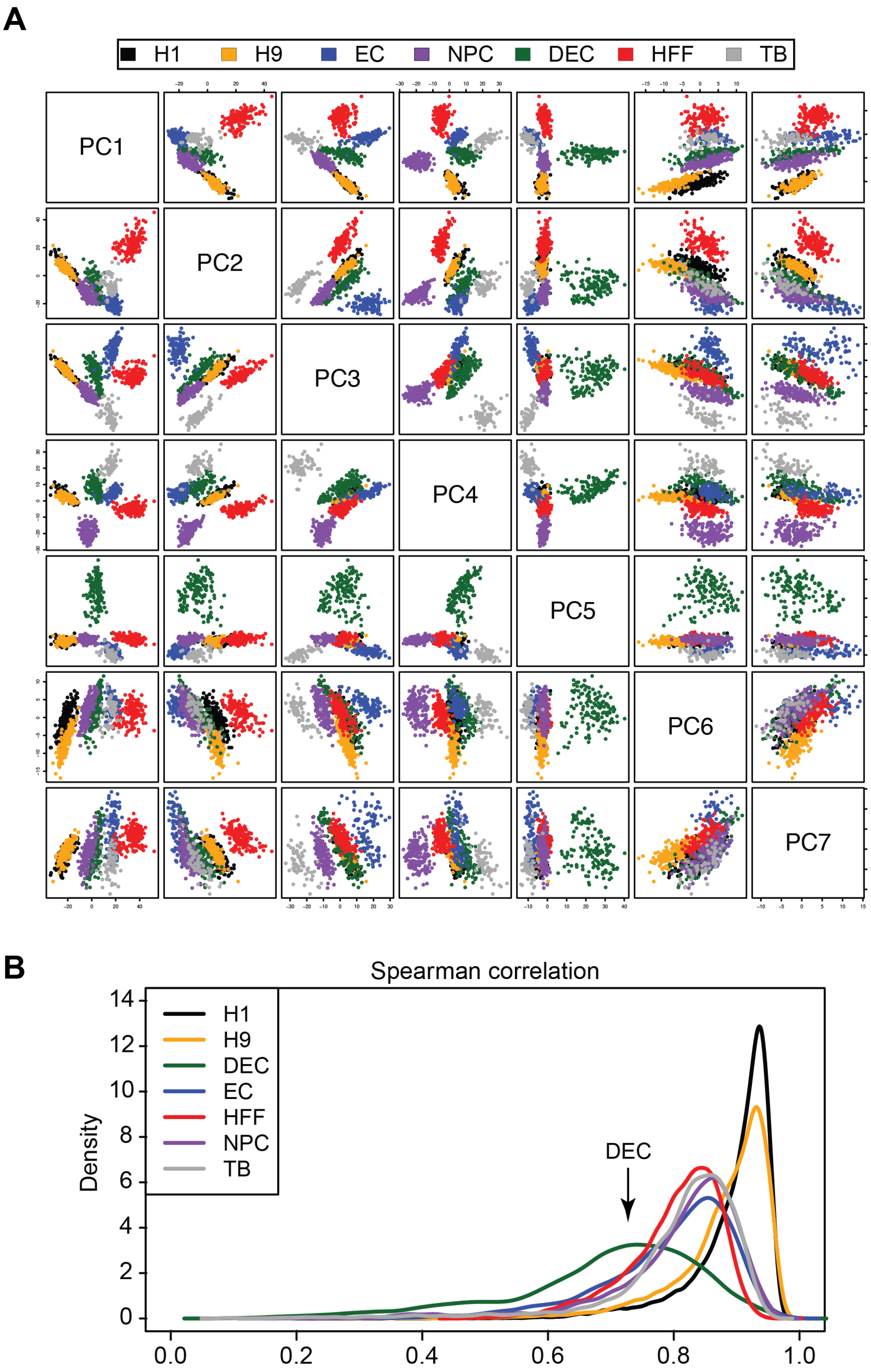

Fig. S2. PCA and heterogeneity analysis of human ES-derived progenitors. a Bulk-projected PCA on progenitor scRNA-seq data, showing from PC1 to PC7. Each cell type is indicated by a unique color. **b** Density plot showing overlay of Spearman correlation within each progenitor cell type. The arrow indicates the lower correlations' distribution in single DECs. NPCs = neuronal progenitor cells. DECs = definitive endoderm cells. ECs = endothelial cells TBs = trophoblast-like cells. HFFs = human foreskin fibroblasts.

Figure S3

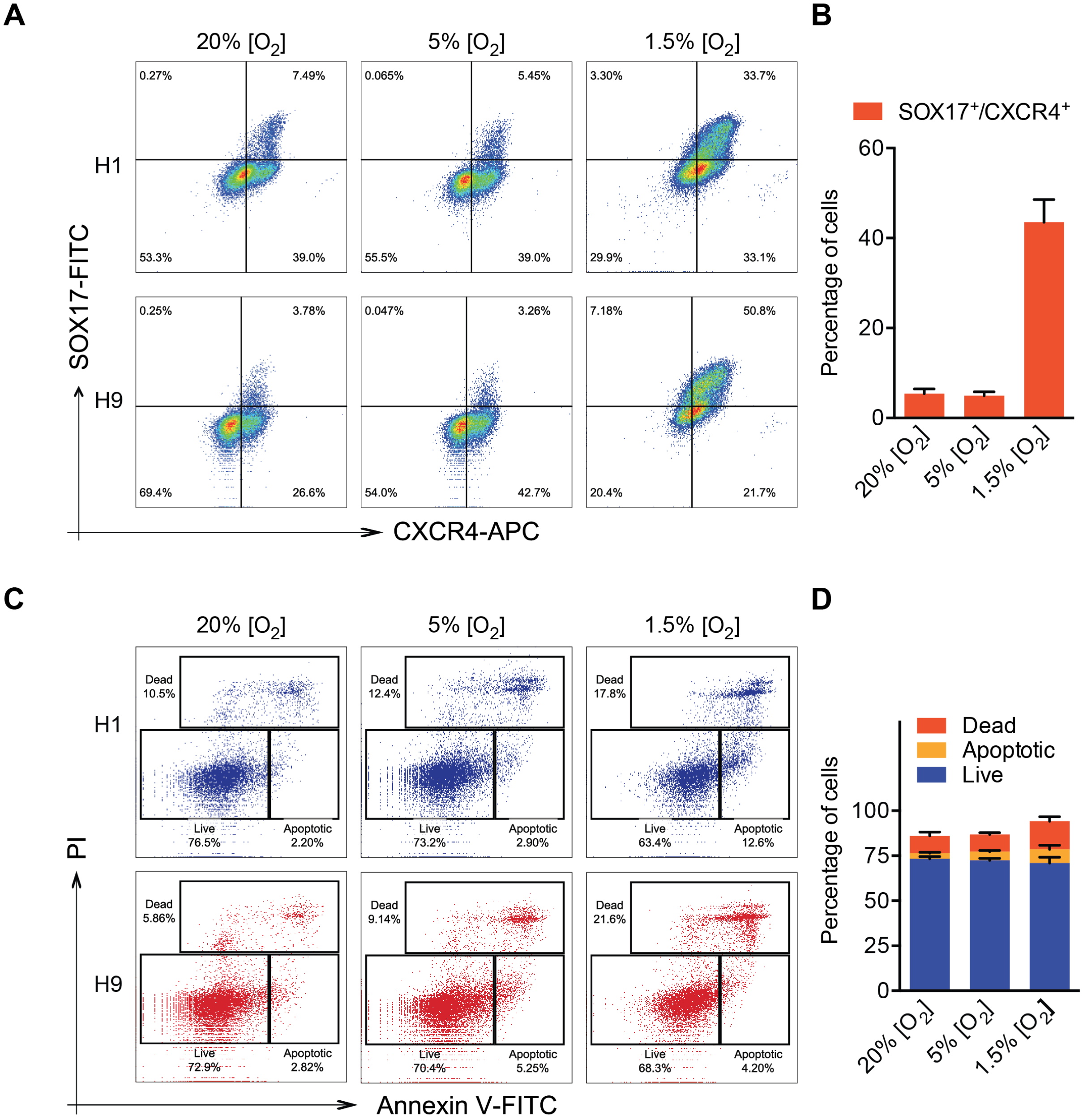

Fig. S3. Characterizations of the impacts of hypoxia on DE differentiation. **a** FACS analysis of definitive endoderm differentiation at day 3 with hypoxia conditions. Cells are co-stained with antibodies against CXCR4 (x axis) and SOX17 (y axis). The percentage of each subpopulation is indicated. Gating is based on undifferentiated controls. **b** Quantifications of CXCR4^{+}/SOX17^{+} double positive cells from three independent experiments similar to **a.** **c** FACS analysis comparing the influence of hypoxia conditions on cell death at day 3 of differentiation. Cells were co-stained with PI (Propidium Iodide, y axis) and Annexin V-FITC (x axis). **d** Quantifications of each populations from two independent experiments similar to **c**. Data is shown as mean ± S.D.

Figure S4

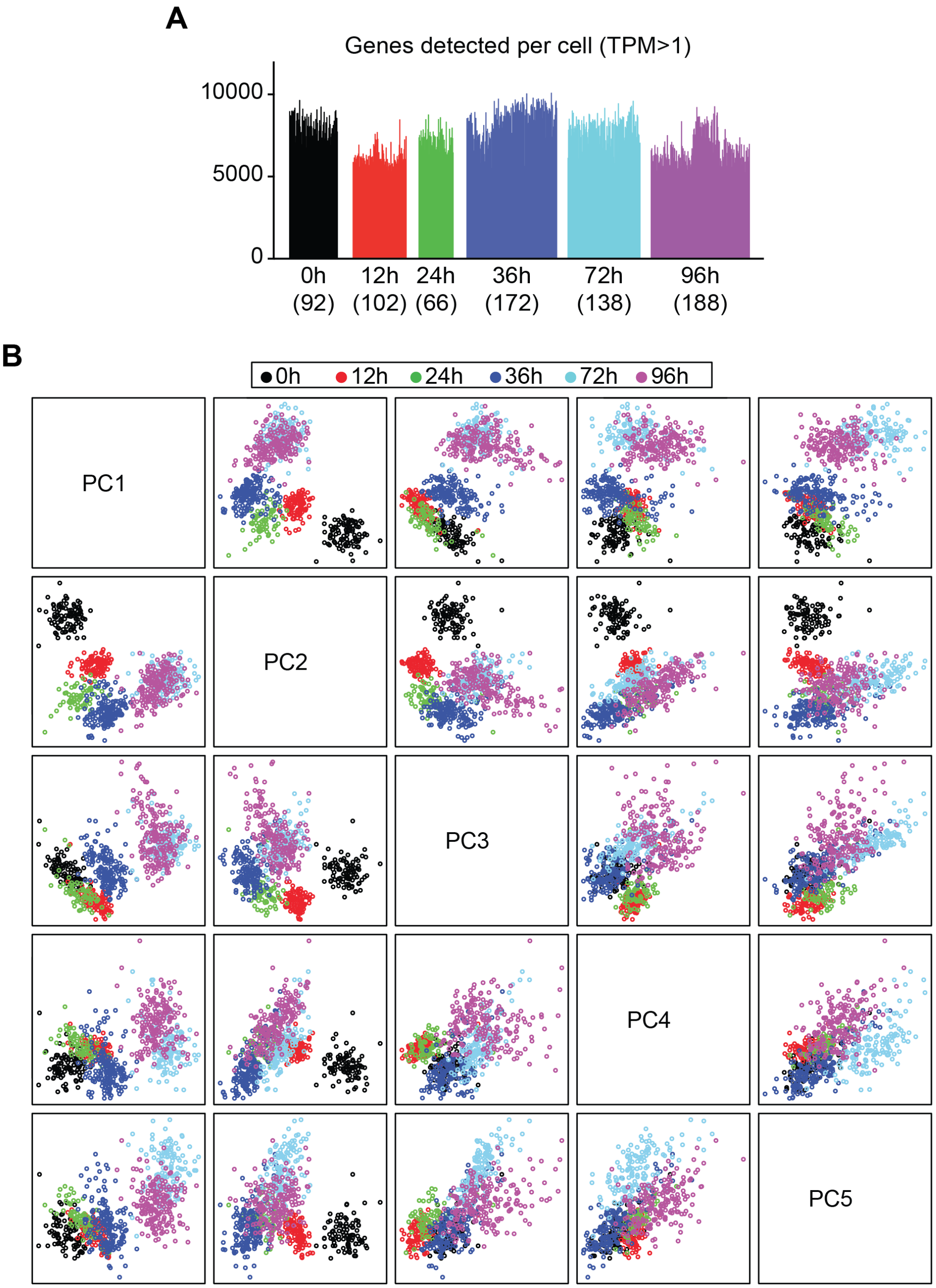

Fig. S4. Quality control and PCA of time course scRNA-seq experiments. a Number of genes detected per cell with TPM > 1. Each individual colored bar represent the data from one cell at indicated time points. Numbers of single cells profiled for each time point are indicated in parentheses. **b** Density plot showing overlay of Spearman correlation within each time point of single cells. **c** Bulk-projected PCA on time course scRNA-seq data, showing from PC1 to PC5. Each time point is indicated by a unique color.

Figure S5

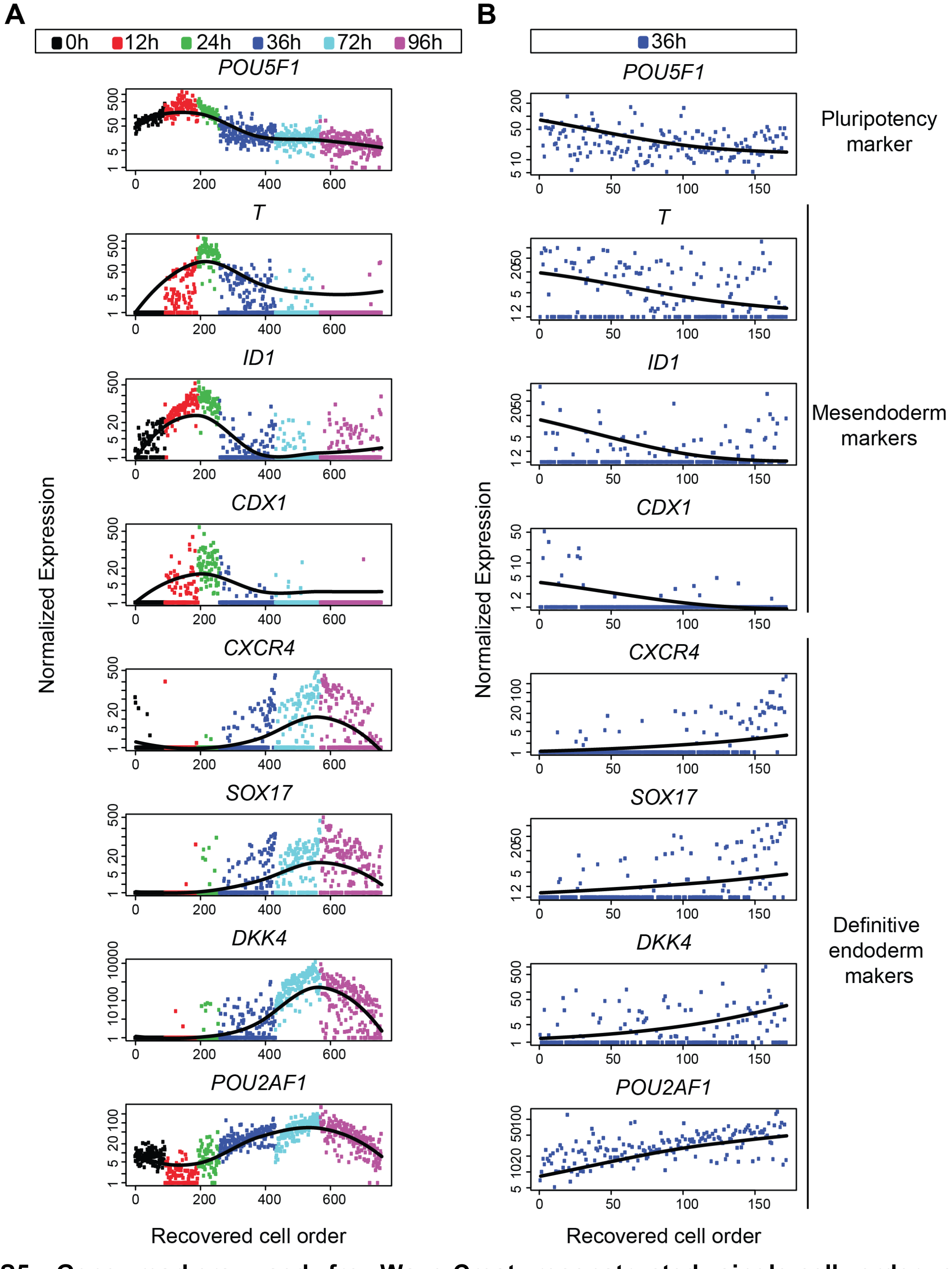

Fig. S5. Gene markers used fro Wave-Crest reconstructed single-cell order of DE differentiation trajectory. a Shown are the eight marker genes used for reconstruction. For comparisons, *POU5F1*, *T*, *CXCR4* and *SOX17* plots are identical to the ones shown in **Fig. 3c**. The y axis shows normalized expression. The x axis shows cells across all the time points, following the Wave-Crest reconstructed order. Fitted lines of gene-specific expression are shown in black. **b** Shown are the same eight genes as in **a**, following the Wave-Crest reconstructed cell order. Instead of including all the cells, only cells from 36 hours are shown here.

Figure S6

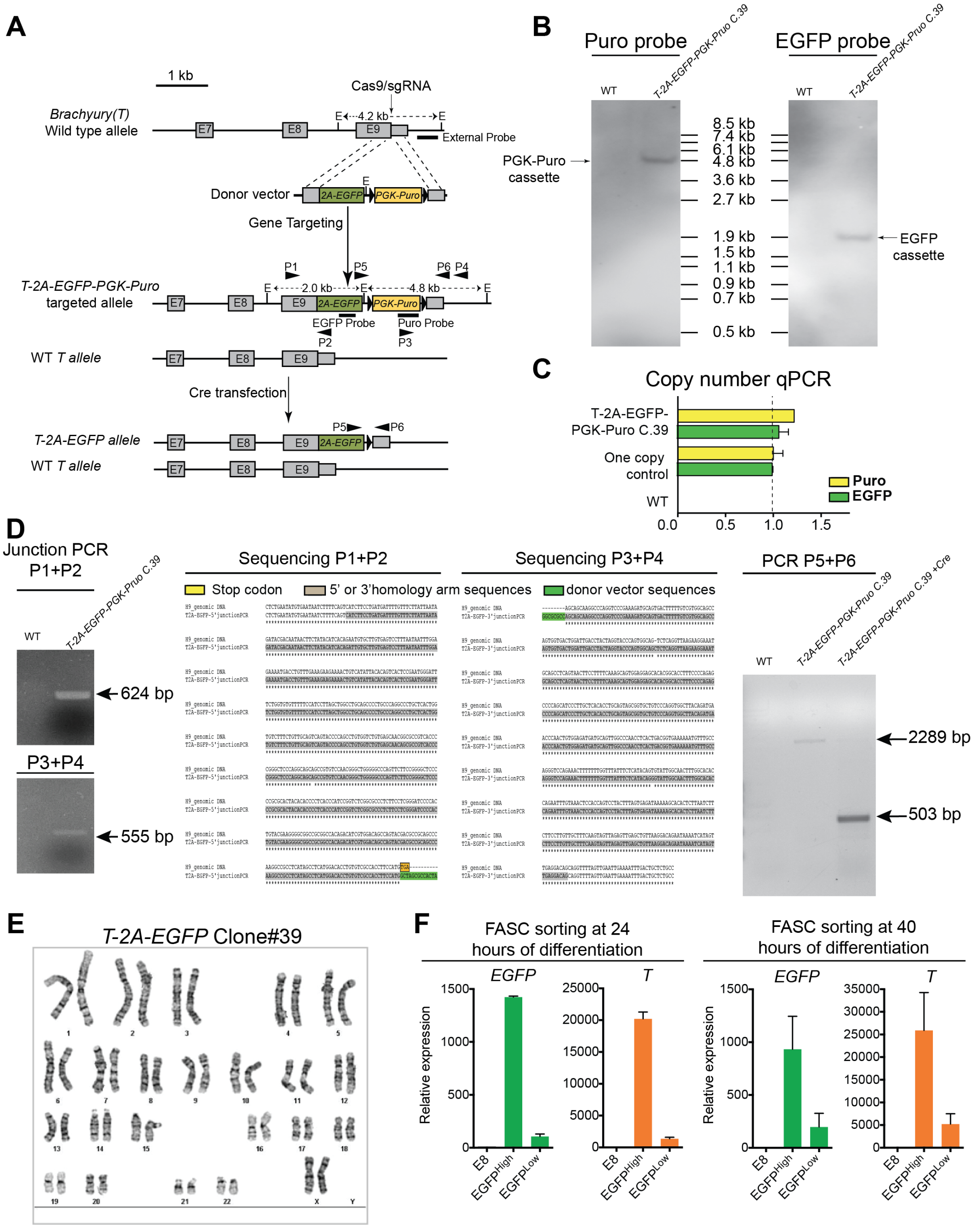

Fig. S6. Characterizations of *T-2A-EGFP* knock-in reporter. a Outline of *T-2A-EGFP-PGK-Puro* knock-in strategy. Gray boxes indicate the exons of the endogenous gene. The arrow indicates the CAS9/sgRNA cut site. Arrowheads indicate the DNA oligos used for junction PCR. **b** Southern blot with internal probes against *EGFP* or *PGK-Puro* cassette. **c** Copy number qPCR analysis confirm *EGFP* or *Puro* cassette knock-in as one copy in the reporter cell line. **d** Junction PCR and alignment to genomic DNA confirm the knock-in cassettes and PCR showing Cre-transfected removal of the *PGK-Puro* cassette. **e** Cytogenetic test on *T-2A-EGFP* line after gene targeting and expansion. **f** qPCR analysis of sorted EGFP^{High} and EGFP^{Low} populations during two time points (24 and 40 hours) of differentiation, confirming the co-expression and enrichment between EGFP and the endogenous *T* gene expression. Relative expression is normalized to GAPDH and undifferentiated ES cells (E8). Data is shown as mean ± S.D.

Figure S7

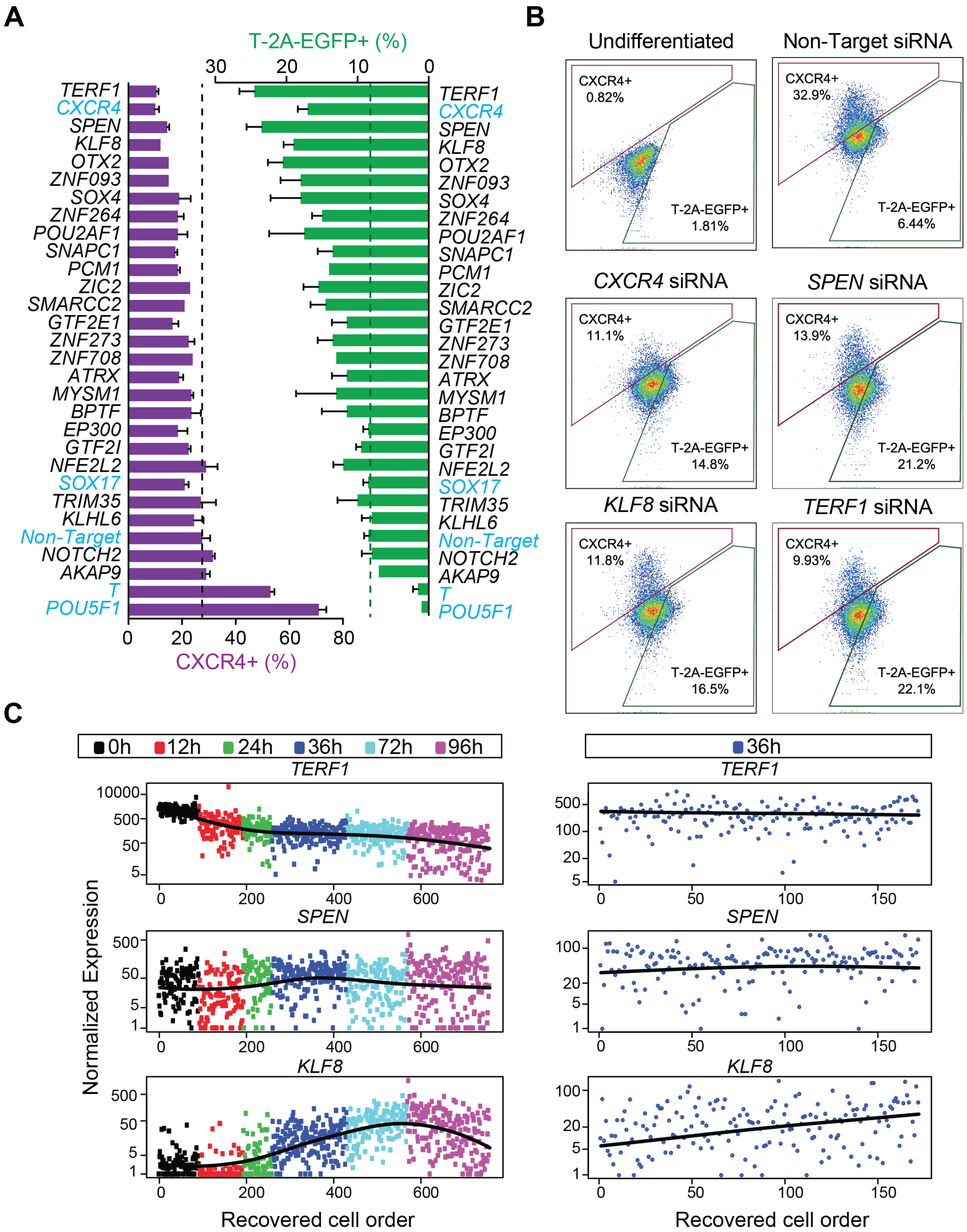

Fig. S7. Summary of siRNA experiments and characterization of top candidate genes.
a Summary of siRNA knockdown experiments showing both the percentages of T-2A-EGFP$^+$ and CXCR4$^+$ cells, ordered as in **Fig. 5b**. Dashed line indicates the levels of cells transfected with the non-target siRNA control. Genes in blue font indicate control experiments. Data is shown as mean ± S.D. **b** FACS analysis of the top three candidate genes (by Differentiation score) in **Fig. 5b**. For comparisons, undifferentiated condition, *Non-Target*, *CXCR4* and *KLF8* siRNA are identical to those shown in **Fig. 5a**. X axis indicates GFP channel, y axis indicates APC channel. **c** Gene expression dynamics over four days (left panel) or at 36 hour (right panel) of differentiation following the Wave-Crest reconstructed cell order as in **Figs. 3c** and **S4**. X axis indicates cells following Wave-Crest recovered order. Y axis indicates normalized expression value. Fitted lines of gene-specific expression are shown in black.

Figure S8

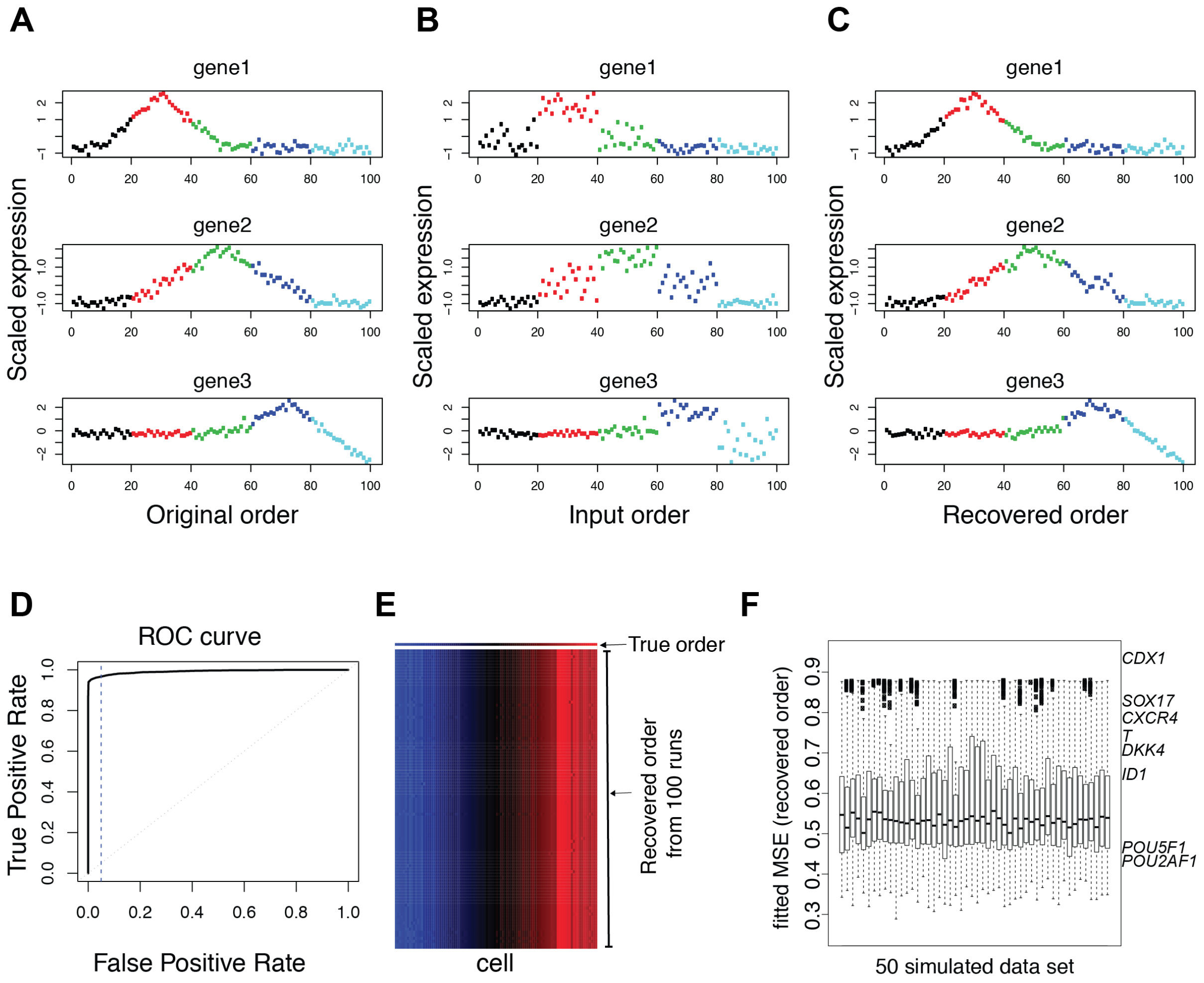

Fig. S8. Simulation results evaluating Wave-Crest.
a Three example genes that are simulated with dynamic trend. The y axis shows rescaled expression. The x axis shows cells following the original simulated order. Cells from different time points are shown in different colors. **b** Shown are the same 3 genes as in **a**. Instead of ordering cells by the original order, in b the cell order within each time point is perturbed. This is also the input cell order to run Wave-Crest. **c** Shown are the same 3 genes as in **a** and **b**. Cells are ordered following Wave-Crest recovered order. **d** ROC curve of Wave-Crest on simulated data sets. The x and y axis shows FPR and TPR averaged over 50 simulations. **e** Wave-Crest reconstructed cell orders using 100 sets of different initial points on one simulated data. The original orders of cells are indicated using different colors following a color gradient from blue to red. The first row of **e** shows cells following the original order. The 100 rows below show cells following the reconstructed order in the 100 Wave-Crest runs with different initial points, respectively. **f** Fitted MSEs of genes identified in each of the 50 simulated data sets. Each box represents one simulated data set. The MSEs of the 8 genes in Figure 3 are marked on the right. MSEs of these 8 genes are calculated using the scRNA-seq data, following the Wave-Crest recovered cell order.

Figure S9

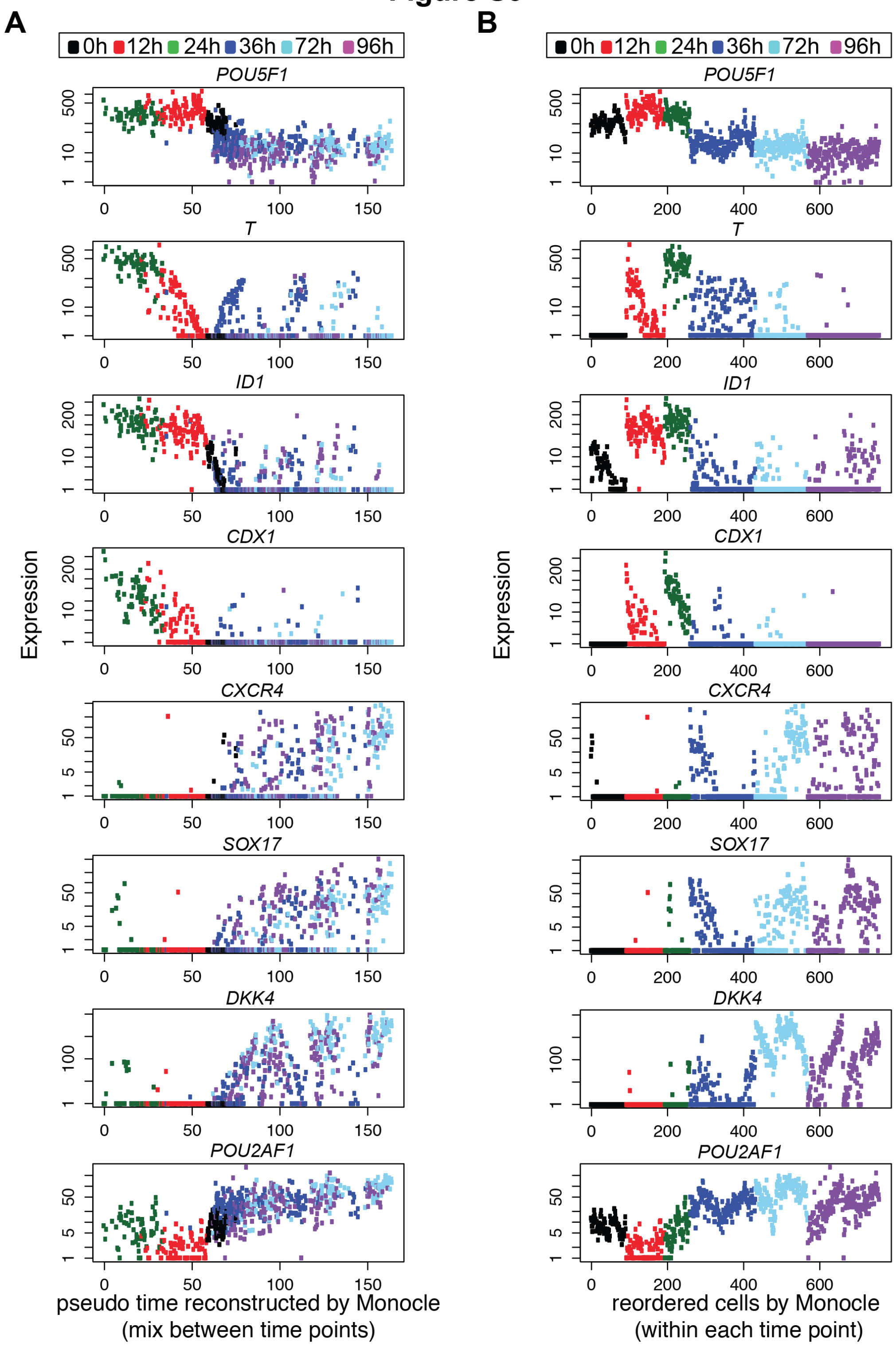

Fig. S9. Monocle results on DE differentiation time course scRNA-seq data.
a Shown are the same 8 genes as in **Fig. 3** and **S5**. The y axis shows normalized expression; the x axis shows cells following monocle recovered pseudo time. Cells from different time points are shown in different colors as indicated at the to of each panel. **b** Shown are the same 8 genes as in **a**. The x axis shows genes following the recovered order by running Monocle within each time point.

Figure S10

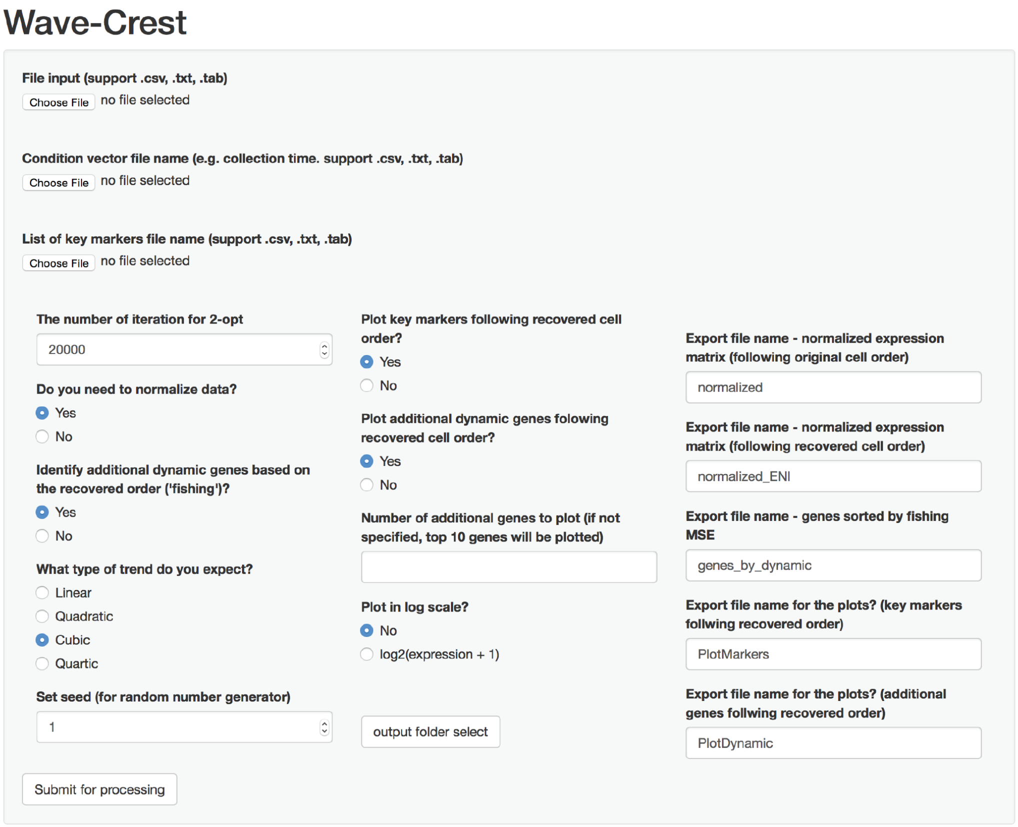

Fig. S10. Screen-shot of Wave-Crest graphical user interface. The Wave-Crest graphical interface is implemented using R/shiny (https://github.com/lengning/WaveCrest). The graphical interface takes scRNA-seq expression file, collection time information, and a group of genes of interest. It outputs recovered cell order and detected genes from 'fishing step'.

SUPPLEMENTARY RESULTS

Evaluation on Simulation studies

We conducted 50 simulations to evaluate the performance of Wave-Crest. In each simulation, we simulated 100 cells from 5 time points. Each time point contains 20 cells. Five hundred genes were simulated in each of the data set. Among the 500, 100 were simulated as genes with dynamic expression patterns. The dynamic genes were simulated as a random kernel signal plus random noises generated from Normal (mean = 0, sd = 0.2). The noise genes were simulated from Normal (mean = 0, sd = 1). A table containing all kernel signals is available as Additional File 10: Table S9. Fig. S8a shows 3 example dynamic genes following the original simulated order. Prior to applying the Wave-Crest, we shuffled the cell order within each time point. Fig. S8b shows the same 3 genes as in Fig. S7a, but following Wave-Crest input cell order. Six genes were used to run the ENI and 2-opt algorithm. Recall that in DE empirical data analysis, the key markers used for reordering were identified by combining SCPattern results and information from prior literature. To deconvolute the SCPattern's key marker detection performance vs. Wave-Crest's reordering and fishing performance, we evaluate SCPattern and Wave-Crest in individual simulations. SCPattern evaluation may be found in a companion study [1]. In this particular simulation, in each run we randomly selected 6 genes from the dynamic genes and used them for the reordering. Fig. S8c shows example reordering results – shown are the same genes as in Fig. S8a and b, but following Wave-Crest ENI recovered cell order. After obtaining the recovered cell order, the fishing step was applied to detect additional genes with dynamic expression trends. The permutation p-value cutoff of the fishing step was set as 0.05. Fig. S8d shows the Receiver Operating Characteristics (ROC) curve for gene detection, summarized over all 50 simulations. The x axis shows False Positive Rate (FPR) and the y axis shows the True Positive Rate (TPR). The average TPR and FDR at the 0.05 p-value cut off are 0.967 and 0.051 (with standard deviation 0.012 and 0.010, also shown as a blue line on Fig. S8c)

We also evaluated the consistency of Wave-Crest cell order recovery by running the ENI and 2-opt step using different initial points. Fig. S8e shows Wave-Crest reconstructed cell orders using 100 sets of different initial points on one simulated data set as described above. The original orders of cells are indicated using different colors following a color gradient from blue to red. The first row in Fig. S8e shows cells following the original order. Recall that before running Wave-Crest ENI and 2-opt, the cell order was perturbed. The 100 rows below show cells following the reconstructed order in the 100 Wave-Crest runs with different initial points, respectively. Note the same cell are shown in an identical color in different runs (rows). The similarity between results from different runs indicates that the Wave-Crest ENI and 2-opt step is robust to the choice of initial point. The mean correlation between these 100 recovered orders is 0.993. In addition, the mean correlation between the original order and the 100 recovered orders is 0.982. This also indicates that all the reconstructed order recovered the original order decently. We also conducted the same evaluation on all of the 50 simulations. Averaging over the 50 simulated data set, the mean correlation across 100 ENI-2opt runs is 0.997 and the mean correlation between the original order and the recovered orders is 0.984. We also compared the MSE distribution of genes identified in the simulated data sets to the MSEs in our empirical scRNA-seq data set (Fig. S8f). The result indicates that the simulated data sets are comparable to the empirical data.

Evaluation on empirical data

We also compared the Wave-Crest ENI to Monocle in cell order recovery on the DE differentiation time course scRNA-seq data. Fig. S9a shows the same 8 markers we used in Fig. 3 and S5, following the Monocle recovered pseudotime on the x axis. Monocle was applied on the entire time course differentiation data by allowing only one end-point state. The cells are colored by the original collecting time. Fig. S9a indicates that cells in pseudotime interval [0, 30] are composed of cells from the 24h collection time; cells in pseudotime interval [30, 60] are composed of cells from the 12h collection time 12h; cells in pseudotime interval [60, 162] are composed of a mixed group of cells from 0h, 36h, 72h and 96h, with no clear separation. Results indicate that the reconstructed

pseudotime interval [0, 60] may present a reversed progression time (24h-12h). However, it is not clear to us how to interpret the cell order in pseudotime interval [60, 162] (especially for the expression trend of gene *T*). It is hard to justify whether the reconstructed pseudotime across 758 cells represents the temporal trend of DE cell differentiation.

We also evaluated results from running Monocle on cells within each time point separately. We applied Monocle within each time point and concatenate the reordered cells together. Fig. S9b shows the same 8 genes as in Fig. 3 and S5. The cells are sorted along the x axis by running Monocle within each time point. The results indicate that by running Monocle independently for cells from different time points, the recovered local trend within each time point may not match the global trend over all time points (e.g. gene *ID1*'s 0h reordering and gene *T*'s 12h reordering). This is likely because when running Monocle within each time point separately, we ignored the information from other time points.

SUPPLEMENTARY METHODS

The Wave-Crest graphical interface implementation

The Wave-Crest graphical user interface (GUI) is implemented using R/shiny [2]. Once software R and associated R packages are installed, a user can launch the Wave-Crest GUI by simply typing these commands in R:

```
library(shiny)
runGitHub('lengning/WaveCrest')
```

The input of the GUI requires the scRNA-seq expression file, collection time information, and a group of genes of interest. Once the input files are provided, Wave-Crest ENI and 2-opt algorithms will be applied to reorder cells based on the markers of interest. The output files contains a gene expression matrix following recovered cell order and expression plots of the key markers following

recovered cell order. The user can also choose whether to run the ‘fishing’ step to identify additional genes. If the ‘fishing’ step is enabled, the GUI will also output detected genes and their expression plots following the recovered cell order.

Heterogeneity analysis

To quantify the heterogeneity of each cell type, we calculated Spearman correlation for all cell pairs within each cell type. Densities of the within cell type correlations are shown in Fig. S2.

Gene ontology (GO) analysis

Gene ontology analyses were performed using R/Allez package [3]. For a given PC *n*, the absolute loadings $|\vec{W}^n|$ are used as input. Gene sets with a size smaller than 2 or larger than 500 were not considered in the analyses. Gene sets with p-value ≤ 0.05 were considered as enriched.

Southern Blots

Genomic DNA of targeted clones was purified using PureGene core kit (Qiagen). Ten micrograms of genomic DNA was digested with EcoRI and then resolved on a 1.0% agarose gel. DIG-labeled DNA probe synthesis, DNA gel transfer, and blot hybridization and visualization were done according to Roche DIG application manual. DNA oligos used to amplify the Southern blot probe are listed in Additional file 8: Table S7.

FACS analysis and cell sorting

Single live cells were stained in cold PBS + 1% FBS (HyClone). All the primary antibodies used to perform FACS sorting or analyses are listed in Additional file 7: Table S6. Cell viability tests was performed using The Dead Cell Apoptosis Kit with Annexin V Alexa Fluor™ 488 & Propidium Iodide (PI) according to the protocols provided by the manufacturer (ThermoFisher Scientific, V13245). FACS was performed on the FACSAria IIIu or FACSCanto II instrument and using

FACSDiva software version 6.1.3 (all from Becton Dickinson). FACS analysis was performed using FlowJo software version X 10.1.

REFERENCE

1. Leng N, Chu, L.F., Choi, J., Kendziorski, C., Thomson, J.A. and Stewart, R.M.: **SCPattern: A statistical approach to identify and classify expression changes in single cell RNA-seq experiments with ordered conditions.** *bioRxiv* 2016.
2. **shiny: Web Application Framework for R** [https://cran.r-project.org/web/packages/shiny/index.html]
3. Newton MA, Quintana FA, Den Boon JA, Sengupta S, Ahlquist P: **Random-Set Methods Identify Distinct Aspects of the Enrichment Signal in Gene-Set Analysis.** *Annals of Applied Statistics* 2007, **1:**85-106.
